# Results of a 15-year systematic survey of commensal rodents in English dwellings

**DOI:** 10.1038/s41598-017-15723-9

**Published:** 2017-11-21

**Authors:** M. Lambert, F. Vial, S. Pietravalle, D. Cowan

**Affiliations:** National Wildlife Management Centre, Animal and Plant Health Agency, Sand Hutton, York, YO41 1LZ UK

## Abstract

Population trends for commensal rodents are the subject of interest and speculation but accurate data are rarely available. Here we report data from a 15-year systematic survey of rats and mice in English dwellings and present national-level estimates of prevalence for 1996–2010. We found evidence for peaks in prevalence of mice inside and rats around dwellings in 2002 and 2008. Models containing twelve variables relating to the dwelling and local area explained some but not all of the variation in prevalence. Older dwellings, those in rural areas, those with litter, drainage faults and pets or other animals outdoors tended to have higher levels of rodent prevalence. Regional differences were found but there were no seasonal trends apart from lower prevalence of mice during summer. Rodent prevalence was generally higher in rented (compared to owner-occupied) dwellings, although apparently not due to reduced tendency to carry out rodent control. The percentage of households having taken some form of action against active rodent problems varied according to prevalence at the national level, and therefore appropriate data on number of rodent control treatments carried out each year could likely act as a useful index of household rodent prevalence.

## Introduction

Rats and mice generate considerable public interest; they evoke fear, repulsion and fascination. Metrics of their close proximity and ratios of their abundance to ours have been the source of much speculation and conjecture, yet paradoxically, there are very few recent reliable data on their abundance and distribution. An explanation perhaps lies in their familiarity. In opportunistic wildlife surveys for example, recorders may be more likely to report species that they are more interested in, thus rare and iconic species may be over represented while common species may be under-reported^1–3^. This problem may be exacerbated for common species with lower detectability, and recorders may be more inclined to overlook species regarded as pests rather than wildlife. Hence, while assumed to be ubiquitous throughout Britain, patchy distribution records for house mice (*Mus musculus)* and Norway rats (*Rattus norvegicus*) have been reported, and it has been suggested that they are ignored because of their familiarity and hence under-recorded^4^. Given the well-documented public and animal health risks associated with commensal rodents, access to reliable information on distribution and trends in abundance is important for developing and monitoring rodent control policy and strategies.

Due to difficulties in obtaining accurate population estimates using traditional sampling methods, presence-absence surveys may be preferable in urban habitats and the potential usefulness of a systematic survey of rodent prevalence in English dwellings was first proposed over 40 years ago^5^. As well as lower likelihood of reporting bias, systematic surveys provide opportunities to collect contextual or environmental data which facilitates investigations of habitat preference and distribution across environmental gradients^6^. Rodent abundance in urban contexts is influenced by land cover type at the landscape scale, socio-economic conditions at the neighbourhood or district level, and dwelling-specific variables at a local level, including availability of food and harbourage for example^7–10^ Using data collected as part of the 1996 English House Condition Survey (EHCS), Langton, Cowan and Meyer^11^ found associations between dwelling-specific variables and prevalence of commensal rodents. Again there was a link with access to availability of food resources (occurrence of Norway rats and house mice was greater for dwellings with animals or pets in the garden) and harbourage; dwellings in areas with substantial problems such as dereliction, litter, vacant properties and unkempt gardens had significantly higher prevalence of rats and mice. All of these relationships potentially provide the basis for an ecological mechanism that drives rodent population change, and potential opportunities for ecologically-based wildlife management methods.

Here we present an analysis of systematic survey data collected in England during the period 1996–2010 to examine temporal patterns (trend and seasonality) in the prevalence of commensal rodents in domestic dwellings, and secondly to explore potential ecological drivers of commensal rodent prevalence in this context.

## Methods

### The surveys

We used data from the EHCS (and subsequently the English Housing Survey; EHS) comprising detailed information on English housing stocks^12^. The 1996 and 2001 EHCS each provided data collected for a sample of approximately 17,000 domestic dwellings during a single year. The surveys used a multi-stage clustered sample stratified from an initial random sample of postcode addresses by region and tenure type to avoid under-sampling of less well-represented tenures; dwellings were not re-sampled between years. Surveys were carried out by a team of approximately 100 surveyors each year with experience in building inspections; they had also received training in identifying signs of rodent activity^11^. The survey included an interview with the householder and visual inspection of the dwelling (including the buildings and any associated plot of land) and also the local area. From 2002 onwards the EHCS was carried out on a continuous rolling programme over successive two-year periods to produce multi-year estimates of housing statistics using a similar approach to the American Community Survey^13^, but otherwise using the same sampling protocols as previously. In 2008 the EHS replaced the EHCS and was designed to ensure maximum continuity with previous surveys^12^. To ‘undo’ the effect of stratified sampling, account for non-responses and ensure that each dwelling contributed correctly to national totals, the EHCS and EHS used grossing factors (dwelling weights)^14,15^. Data for 2002–2009 were received in overlapping two-year data sets (2002 + 2003, 2003 + 2004…) with associated dwelling weights.

#### Rodent prevalence

We looked at records of the presence/absence of 1) mice inside, 2) rats inside and 3) rats outside dwellings separately, i.e. three different categories of rodent prevalence. Dwellings with mice inside were identified as those where either (during the interview element of the survey) the householder said that there was a current problem and it was in the home, or dwellings where the surveyor saw physical evidence of mice in the living room, kitchen, bedroom(s), bathroom or circulation (hallway, stairs, landing)^11^. Dwellings with rats inside were those where the householder said that there was a current rat problem and it was in the home, or dwellings where the surveyor found evidence of rats in the living room, kitchen, bedroom(s), bathroom or circulation. Dwellings with rats outside were those where the householder said there was a current problem and it was in the garden, or dwellings where the surveyor found evidence of rats within the garden (i.e. privately owned plot associated with the dwelling). Evidence of mice living outdoors were not considered in the analyses; this was because house mice are infrequently found outside buildings in the United Kingdom (UK), particularly in urban areas, and signs of mice are more difficult to reliably detect outdoors in these contexts^11,16,17^. We excluded vacant dwellings, as they have different profiles of rodent presence, in addition, householder interview data was not usually available for these properties^18,19^. Dwellings with no private plot were also excluded when considering occurrence of rats around the dwelling but we included all other property categories. Weighted prevalence data (national-level estimates) are summarised in Supplementary Tables S1, S2 and S3; weighted and un-weighted estimates of rodent prevalence were calculated for each EHCS and EHS reporting period. Thereafter, all statistical analyses were performed on un-weighted data (sample-level estimates) using R^20^ after removing repeated records by excluding alternate two-year data sets.

### Statistical analyses

#### Temporal patterns in rodent prevalence

In the first instance, models investigating the effect of year and month of survey on rodent prevalence were fitted. No survey data were collected from 1997 to 2000. Because of the lack of rodent presence data for this period, only data collected post-2001 were included in the analyses. Fifteen different models were fitted for each rodent prevalence category (see Supplementary Tables S4, S5 and S6). Eight models were generalized linear models (GLM) fitted with binomial errors and logit link function to model the effects of year and month of survey (with both treated either as factors or covariates) on the un-weighted probability of rodent being present; seven were generalized additive models (GAMs) fitted with binomial errors and logit link function to model the effects of year and month of survey as a smoothed function, using thin plate regression splines^21^, on the un-weighted probability of rodents being present. GAMs were fitted using the R package mgcv^22^. The best model for each rodent prevalence category was defined as the model with the lowest Akaike Information Criterion (AIC) and a significant reduction in model deviance (Chi-square test for analysis of deviance) compared to the null model.

#### Factors associated with rodent prevalence

Factors associated with rodent prevalence are potential drivers of population change. Data for a large number of variables were collected in the surveys; thirteen factors were chosen for analysis, either because they had been shown by Langton, Cowan and Meyer^11^ to be related to occurrence of commensal rodents, or they were thought *a priori* to affect the probability of rodents being reported by householders. The 13 candidate factors were litter around the dwelling, plot width, pets/livestock kept outside, situation of block, drainage system faults, tenure type, dwelling type, date of construction, region, number of dwellings in area, nature of area, problems in area, and whether there were any current or previous rodent control arrangements in place. Further details are given in Supplementary Methods. In initial data exploration we looked for any systematic changes in recording effort that could lead to bias in un-weighted survey data; an increasing tendency to sample dwelling categories linked with greater rodent prevalence for example could potentially lead to spurious results. Incomplete records (with at least one missing value) were removed in order for model comparison to be performed (i.e. all models were fitted to exactly the same restricted dataset). Variables were added one at a time (forward selection), starting from a base model containing the temporal variables (year and/or month of survey) in the form identified as best fit in the models fitted above. Model variables were only retained if they resulted in a lower model AIC and a significant reduction in model deviance (Chi-square test for analysis of deviance). The impact of collinearity between variables on the precision of the estimate of a coefficient was evaluated using the variance inflation factor (VIF) using a threshold of VIF >3 to identify and remove collinear variables^23^. Collinearity arises from dependencies between factors; it has previously been suggested for example that rodent control activities and tenure may be linked such that owner occupiers may be more likely to carry out rodent control than occupants of rented properties^24^. The area under the receiver operating characteristic (ROC) curve, AUC, was computed as a measure of the final models’ accuracy.

## Results

### Rodent prevalence

**Figure 1:**
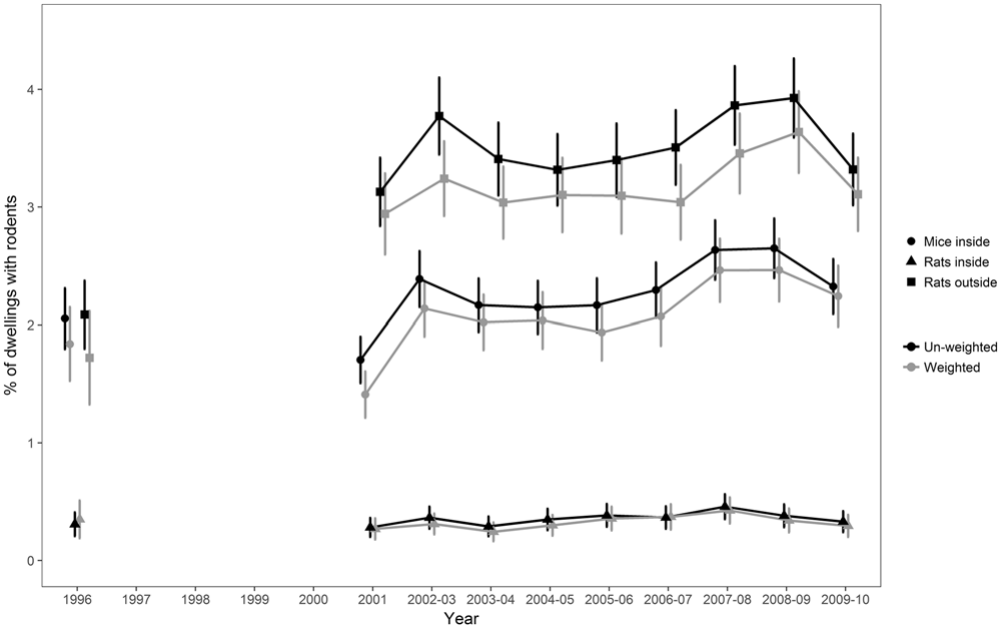
Mean un-weighted prevalence of (**A**) mice inside, (**B**) rats inside and (**C**) rats outside dwellings in each survey; data for 2002–2010 are multi-year estimates (refer to Supplementary Tables S1 and S2 for sample sizes). Weighted prevalence data are shown for comparison. Error bars represent the 95% confidence interval around the mean.

The full data set (with repeated survey data) contained 162,804 records; once repeated data were removed 99,712 records remained. The un-weighted prevalence of mice varied between 1.70% and 2.65%, prevalence of rats inside properties varied between 0.28% and 0.46% and prevalence of rats around dwellings varied between 2.09% and 3.92% (Supplementary Tables S1 and S2). Weighted and un-weighted prevalence followed a similar pattern (Fig. 1).

### Change in rodent prevalence over time

**Figure 2:**
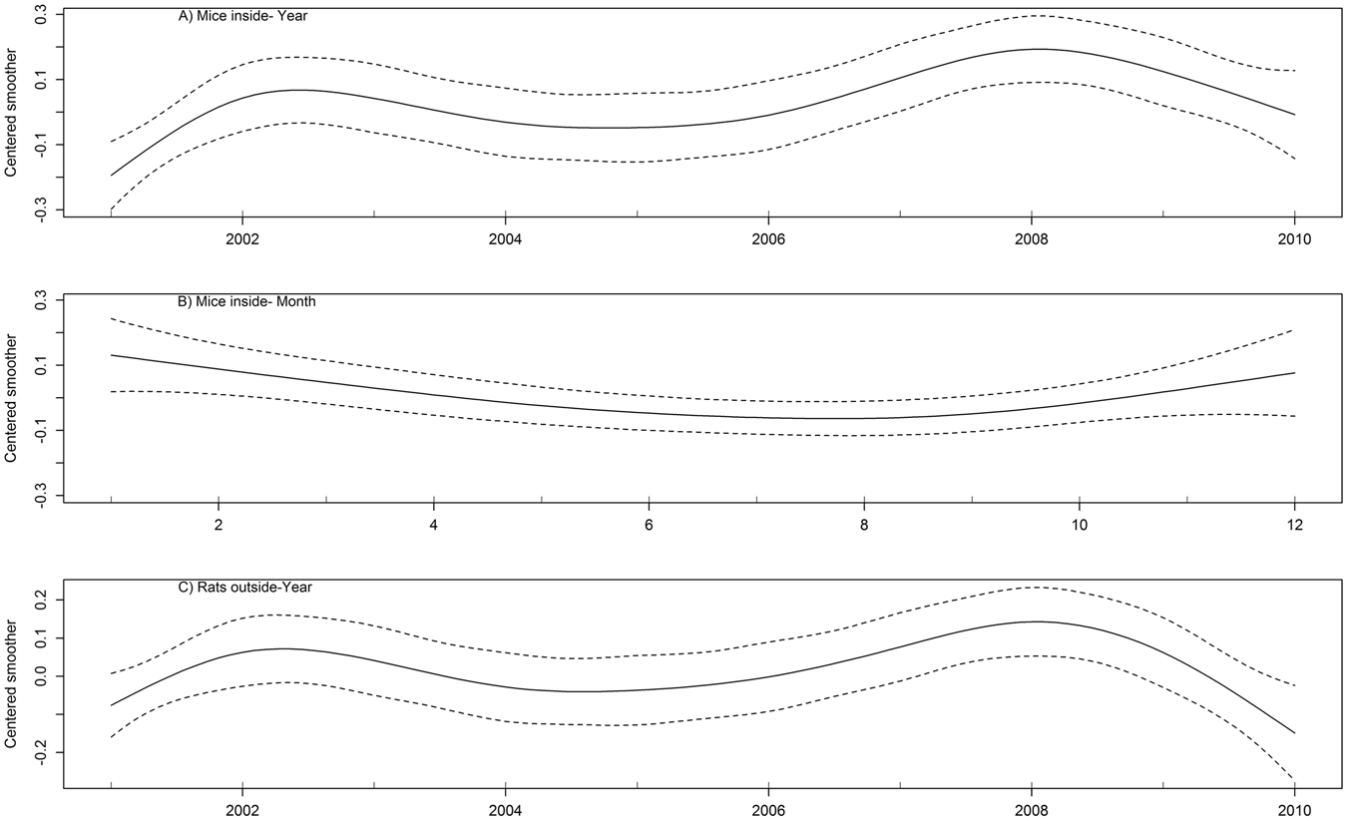
Annual trends in prevalence of commensal rodents in and around English dwellings for the continuous data set (2001–2010); prevalence of mice inside dwellings varied by (**A**) year and (**B**) month of survey, prevalence of rats around dwellings (**C**) varied by year of survey only. There were no significant temporal trends in prevalence of rats inside dwellings. Estimated smoothers (solid line) were fitted by generalized additive modelling with 95% confidence bands shown by dashed lines.

**Figure 3:**
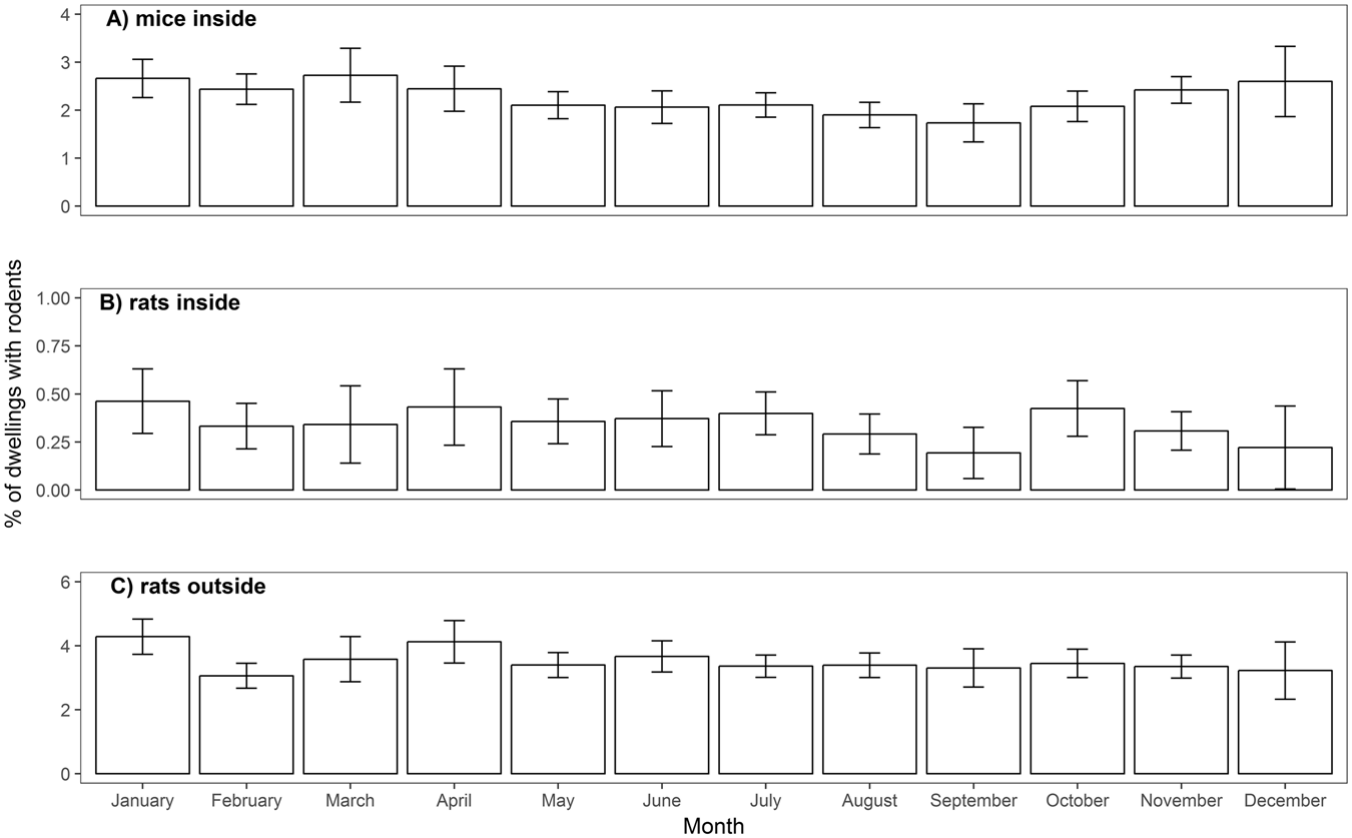
Mean un-weighted prevalence of (**A**) mice inside, (**B**) rats inside and (**C**) rats outside dwellings in different survey months between 2001 and 2010. Error bars represent the 95% confidence interval around the mean.

The model selection process suggested the use of generalized additive models (GAMs) fitted with a smooth function to model the effects of year and month of survey on the un-weighted probability of mice inside and rats being present outside dwellings respectively (Supplementary Tables S4 and S6). There were two distinct peaks in prevalence with a higher probability of having mice inside and rats outside dwellings in 2002 and 2008 than in other years (Fig. 2). Mice were less frequently reported inside dwellings in the warmer months (Fig. 3); excluding month of survey provided a marginally better (ΔAIC < 2) model fit for prevalence of rats outside  dwellings (Supplementary Table S6). Year and month of survey had no statistically significant effect on the reported prevalence of rats inside dwellings (Supplementary Table S5).

### Factors associated with rodent prevalence

Information on whether rodent control arrangements had been or were currently in place was recorded for 5,837 dwellings. Rodent control had been carried out in 2,470 of 3,258 (75.8%) dwellings where there was a current rodent problem; there appeared to be no consistent differences between tenure types in the proportion of affected households taking action against rodent problems (Figure S6). Surveyors had been asked to record information on rodent control activities only for dwellings with a current rodent problem; it was not recorded for the majority of dwellings and was therefore not included as a factor in the final models. However for dwellings with a current rodent problem at the time of the survey, generalized additive models indicated that the proportion having done something about the problem varied over time (df = 8.34, deviance, 30.58, p < 0.001) as did the proportion of dwellings taking action at the time of the survey (df = 8.49, deviance, 79.42, p < 0.001); to some extent these trends matched trends in rodent prevalence (Figure S7).

After removing cases with other missing data, the restricted dataset used for the mice inside and rats outside dwellings models comprised 68,828 records, of which 1,615 reported the presence of mice inside and 257 the presence of rats inside the dwellings. The final dataset for the rats outside dwellings model was made up of 68,812 records (households without a private plot were excluded), of which 1,535 reported the presence of rats outside the dwellings. Variance Inflation Factor (VIF) values were below 3 indicating independence between the candidate explanatory variables (Supplementary Table S7). Proportions of dwellings within each survey category remained relatively stable over time (Figure S5) indicating a lack of survey bias. Model fit for prevalence of mice inside, rats inside and rats outside (AUC of 0.76, 0.75 and 0.73 respectively) was fair^25^ (ROC curves shown in Supplementary Figure S1). We found evidence that the effects of some variables on rodent prevalence varied between different regions, for example in most regions prevalence of mice was highest for privately rented dwellings but this was not the case in the East Midlands and Eastern regions where prevalence was higher in local authority rentals. Significant interactions are illustrated in Supplementary Figures S2–S4; odds ratios (OR) for the main effects are reported in Tables 1–3 in order to draw generalisable patterns in rodent prevalence in English dwellings, estimates of rodent prevalence for each factor level are shown in Table 4 (the OR are used to compare the relative odds of the occurrence of the outcome of interest (e.g. mice inside dwellings) given exposure to the variables of interest (e.g. drainage system faults, tenure type)).

**Table 1:** Results of the multivariate GAM for the presence of mice inside dwellings. Odds ratio (OR) and 95% confidence intervals are presented for the variables retained in the final model.

Parametric coefficients	Odds Ratio	95% Confidence Interval	p value
Litter (No)	—		<0.001
Litter (Yes)	2.03		
Number of dwellings in area (500+)			<0.001
Number of dwellings in area (300–499)	0.75	0.60–0.94	
Number of dwellings in area (100–299)	0.83	0.70–1.00	
Number of dwellings in area (50–99)	0.83	0.68–1.01	
Number of dwellings in area (25–49)	0.88	0.70–1.09	
Number of dwellings in area (Less than 25)	1.33	1.05–1.68	
Width of plot (Same as dwelling)	—		<0.001
Width of plot (less than 10 m)	0.87	0.73–1.04	
Width of plot (less than 20 m)	1.03	0.85–1.25	
Width of plot (20 m or more)	1.55	1.21–1.99	
Animals outside (No)	—		0.003
Animals outside (Yes)	1.30	1.10–1.54	
Road type (Major trunk road or main road)	—		0.003
Road type (Side road)	1.00	0.86–1.15	
Road type (Cul de sac/cresent)	0.81	0.68–0.96	
Road type (Private road)	1.13	0.86–1.49	
Road type (No road)	1.33	0.98–1.80	
Nature of area (City centre)	—		<0.001
Nature of area (Urban)	1.47	1.04–2.08	
Nature of area (Suburban residential)	1.00	0.70–1.42	
Nature of area (Rural residential)	1.78	1.21–2.61	
Nature of area (Village centre)	1.95	1.29–2.96	
Nature of area (Rural)	3.06	2.01–4.64	
Drainage system faults (No)	—		<0.001
Drainage system faults (Yes)	1.97	1.59–2.45	
Problems in area (No problems)	—		<0.001
Problems in area (Slight problems)	1.28	1.09–1.52	
Problems in area (Moderate problems)	1.42	1.16–1.73	
Problems in area (Substantial problems)	1.82	1.46–2.28	
Tenure type (Owner occupied)	—		<0.001
Tenure type (Private rented)	1.31	1.13–1.51	
Tenure type (Local authority)	1.84	1.58–2.14	
Tenure type (Housing association (RSL))	1.42	1.18–1.72	
Dwelling type (End terrace)	—		<0.001
Dwelling type (Mid terrace)	1.02	0.85–1.22	
Dwelling type (Semi-detached)	0.73	0.61–0.87	
Dwelling type (Detached)	1.06	0.85–1.32	
Dwelling type (Purpose built flat)	0.72	0.55–0.94	
Dwelling type (Other)	0.74	0.54–1.01	
Date of construction (Pre 1850)	—		<0.001
Date of construction (1850–1899)	0.72	0.57–0.91	
Date of construction (1900–1918)	0.69	0.55–0.88	
Date of construction (1919–1944)	0.59	0.47–0.74	
Date of construction (1945–1964)	0.43	0.34–0.55	
Date of construction (1965–1980)	0.38	0.30–0.49	
Date of construction (Post 1980)	0.33	0.25–0.43	
Region (East Midlands)	—		<0.001
Region (Eastern)	0.81	0.64–1.04	
Region (London)	3.08	2.49–3.81	
Region (North East)	0.95	0.71–1.25	
Region (North West & Merseyside)	0.93	0.74–1.17	
Region (South East)	1.02	0.82–1.28	
Region (South West)	0.70	0.54–0.89	
Region (West Midlands)	0.96	0.76–1.23	
Region (Yorkshire & Humberside)	1.23	0.99–1.54	

The p-value refers to the Chi-square test for analysis of deviance (model with factor included compared to model without the factor). The variable month of survey was not retained in the final model. The smoothed term year had an approximate significance of p < 0.001 but OR for this non-linear term are not presented.

**Table 2:** Results of the multivariate GLM for the presence of rats inside dwellings. Odds ratio (OR) and 95% confidence intervals are presented for the variables retained in the final model.

Parametric coefficients	Odds Ratio	95% Confidence Interval	p value
Litter (No)	—		<0.001
Litter (Yes)	2.18	1.67–2.86	
Width of plot (Same as dwelling)	—		0.03
Width of plot (less than 10 m)	0.52	0.30–0.88	
Width of plot (less than 20 m)	0.66	0.41–1.07	
Width of plot (20 m or more)	0.81	0.50–1.32	
Animals outside (No)	—		0.01
Animals outside (Yes)	1.62	1.11–2.30	
Nature of area (City centre)	—		0.001
Nature of area (Urban)	2.16	0.89–7.13	
Nature of area (Suburban residential)	1.85	0.75–6.16	
Nature of area (Rural residential)	3.23	1.22–11.18	
Nature of area (Village centre)	3.23	1.13–11.63	
Nature of area (Rural)	5.63	2.04–20.03	
Drainage system faults (No)	—		0.006
Drainage system faults (Yes)	2.10	1.26–3.29	
Problems in area (No problems)	—		0.002
Problems in area (Slight problems)	0.92	0.63–1.35	
Problems in area (Moderate problems)	1.17	0.74–1.87	
Problems in area (Substantial problems)	1.97	1.20–3.26	
Tenure type (Owner occupied)	—		0.01
Tenure type (Private rented)	1.36	0.97–1.88	
Tenure type (Local authority)	1.16	0.77–1.72	
Tenure type (Housing association (RSL))	1.92	1.29–2.81	
Date of construction (Pre 1850)	—		<0.001
Date of construction (1850–1899)	0.56	0.35–0.96	
Date of construction (1900–1918)	0.46	0.26–0.79	
Date of construction (1919–1944)	0.45	0.27–0.76	
Date of construction (1945–1964)	0.24	0.14–0.42	
Date of construction (1965–1980)	0.22	0.13–0.40	
Date of construction (Post 1980)	0.19	0.10–0.36	
Region (East Midlands)	—		0.002
Region (Eastern)	1.31	0.70–2.50	
Region (London)	2.69	1.54–4.92	
Region (North East)	0.85	0.36–1.88	
Region (North West & Merseyside)	1.44	0.81–2.66	
Region (South East)	1.43	0.80–2.66	
Region (South West)	1.71	0.96–3.16	
Region (West Midlands)	2.01	1.14–3.71	
Region (Yorkshire & Humberside)	1.09	0.57–2.10	

The p-value refers to the Chi-square test for analysis of deviance (model with factor included compared to model without the factor). The variables year of survey, month of survey, type of dwellings and type of roads were not retained in the final model.

**Table 3:** Results of the multivariate GAM for the presence of rats outside dwellings. Odds ratio (OR) and 95% confidence intervals are presented for the variables retained in the final model.

Parametric coefficients	Odds Ratio	95% Confidence Interval	p value
Litter (No)	—		<0.001
Litter (Yes)	1.83	1.66–2.00	
Number of dwellings in area (500+ )	—		<0.001
Number of dwellings in area (300–499)	1.08	0.90–1.31	
Number of dwellings in area (100–299)	0.86	0.73–1.01	
Number of dwellings in area (50–99)	0.93	0.79–1.10	
Number of dwellings in area (25–49)	1.09	0.90–1.30	
Number of dwellings in area (Less than 25)	1.41	1.16–1.72	
Width of plot (Same as dwelling)	—		<0.001
Width of plot (less than 10 m)	1.13	1.00–1.26	
Width of plot (less than 20 m)	1.21	1.07–1.37	
Width of plot (20 m or more)	1.50	1.26–1.78	
Animals outside (No)	—		<0.001
Animals outside (Yes)	2.77	2.48–3.10	
Road type (Major trunk road or main road)	—		0.007
Road type (Side road)	0.82	0.73–0.93	
Road type (Cul de sac/cresent)	0.88	0.76–1.00	
Road type (Private road)	0.78	0.61–0.99	
Road type (No road)	1.09	0.84–1.42	
Nature of area (City centre)	—		<0.001
Nature of area (Urban)	1.11	0.80–1.54	
Nature of area (Suburban residential)	0.97	0.70–1.35	
Nature of area (Rural residential)	1.64	1.17–2.31	
Nature of area (Village centre)	2.07	1.44–2.97	
Nature of area (Rural)	3.24	2.24–4.69	
Drainage system faults (No)	—		<0.001
Drainage system faults (Yes)	1.73	1.43–2.10	
Problems in area (No problems)	—		<0.001
Problems in area (Slight problems)	1.35	1.19–1.54	
Problems in area (Moderate problems)	1.78	1.52–2.09	
Problems in area (Substantial problems)	2.65	2.21–3.18	
Tenure type (Owner occupied)	—		<0.001
Tenure type (Private rented)	0.98	0.86–1.12	
Tenure type (Local authority)	1.50	1.33–1.69	
Tenure type (Housing association (RSL))	1.32	1.14–1.53	
Date of construction (Pre 1850)	—		<0.001
Date of construction (1850–1899)	0.71	0.58–0.88	
Date of construction (1900–1918)	0.84	0.67–1.04	
Date of construction (1919–1944)	0.75	0.62–0.92	
Date of construction (1945–1964)	0.52	0.43–0.64	
Date of construction (1965–1980)	0.51	0.41–0.63	
Date of construction (Post 1980)	0.53	0.43–0.66	
Region (East Midlands)	—		<0.001
Region (Eastern)	0.95	0.79–1.14	
Region (London)	1.00	0.82–1.22	
Region (North East)	0.96	0.77–1.19	
Region (North West & Merseyside)	0.84	0.70–1.01	
Region (South East)	1.00	0.84–1.19	
Region (South West)	1.02	0.85–1.23	
Region (West Midlands)	1.77	1.50–2.10	
Region (Yorkshire & Humberside)	1.05	0.88–1.25	

The p-value refers to the Chi-square test for analysis of deviance (model with factor included compared to model without the factor). The variables month of survey and type of dwellings were not retained in the final model. The smoothed term year had an approximate significance of p < 0.001 but OR for this non-linear term are not presented.

**Table 4:** Number and percentage of occupied dwellings with rodents present by factor type. Un-weighted percentages are presented (2001–2010).

	Sample	Mice inside		Rats inside		Sample	Rats outside	
		n	Un-weighted %	n	Un-weighted %		n	Un-weighted %
**(a) Tabulated by litter**								
No	63784	1056	1.66 (1.56–1.75)	160	0.25 (0.21–0.29)	54343	1448	2.66 (2.53–2.80)
Yes	22473	878	3.91 (3.65–4.16)	147	0.65 (0.55–0.76)	17197	1063	6.18 (5.82–6.54)
**(b) Tabulated by number of dwellings in area**								
500+	9172	252	2.76 (2.42–3.09)	29	0.32 (0.20–0.43)	7031	246	3.50 (3.07–3.93)
300–499	9335	189	2.02 (1.74–2.31)	31	0.33 (0.22–0.45)	7304	254	3.48 (3.06–3.90)
100–299	27667	543	1.97 (1.80–2.13)	73	0.26 (0.20–0.32)	22276	569	2.55 (2.35–2.76)
50–99	19396	339	1.75 (1.56–1.93)	63	0.32 (0.24–0.40)	16290	451	2.77 (2.52–3.02)
25–49	12724	220	1.73 (1.50–1.96)	37	0.29 (0.20–0.38)	11085	374	3.37 (3.04–3.71)
Less than 25	9620	414	4.30 (3.90–4.71)	78	0.81 (0.63–0.99)	8845	631	7.13 (6.60–7.67)
**(c) Tabulated by width of plot**								
Same as dwelling	25864	746	2.88 (2.68–3.09)	125	0.48 (0.40–0.57)	25847	809	3.13 (2.92–3.34)
Less than 10 m	20479	341	1.67 (1.49–1.84)	48	0.23 (0.17–0.30)	20479	635	3.10 (2.86–3.34)
Less than 20 m	20589	341	1.66 (1.48–1.83)	51	0.25 (0.18–0.32)	20589	666	3.23 (2.99–3.48)
20 m or more	4969	257	5.17 (4.56–5.79)	46	0.93 (0.66–1.19)	4967	809	7.53 (6.80–8.26)
**(d) Tabulated by keeping pets outside**								
No	82584	1734	2.10 (2.00–2.20)	263	0.32 (0.28–0.36)	67653	1953	2.89 (2.76–3.01)
Yes	4877	204	4.18 (3.62–4.74)	44	0.90 (0.64–1.17)	4783	533	11.14 (10.25–12.03)
**(e) Tabulated by road type**								
Major trunk road or main road	11999	326	2.72 (2.43–3.01)	59	0.49 (0.37–0.62)	9002	455	5.05 (4.60–5.51)
Side road	38492	994	2.58 (2.42–2.74)	142	0.37 (0.31–0.43)	32418	1073	3.31 (3.12–3.50)
Cul de sac/crescent	33393	469	1.40 (1.28–1.53)	76	0.23 (0.18–0.28)	28298	796	2.81 (2.62–3.01)
Private road	2822	95	3.37 (2.70–4.03)	23	0.82 (0.48–1.15)	2029	106	5.22 (4.26–6.20)
Unmade/no road	1258	74	5.88 (4.58–7.18)	11	0.87 (0.36–1.39)	1123	96	8.55 (6.91–10.18)
**(f) Tabulated by nature of area**								
City centre	2894	80	2.76 (2.17–3.36)	11	0.38 (0.16–0.60)	1298	51	3.93 (2.87–4.99)
Urban	18584	636	3.42 (3.16–3.68)	89	0.48 (0.38–0.58)	13189	471	3.57 (3.26–3.89)
Suburban residential	50677	719	1.42 (1.32–1.52)	114	0.22 (0.18–0.27)	43476	1096	2.52 (2.37–2.67)
Rural residential	10035	210	2.09 (1.81–2.37)	35	0.35 (0.23–0.46)	9438	389	4.12 (3.72–4.52)
Village centre	3352	98	2.92 (2.35–3.49)	17	0.51 (0.27–0.75)	3096	175	5.65 (4.84–6.47)
Rural	2429	216	8.89 (7.76–10.02)	45	1.85 (1.32–2.39)	2376	343	14.44 (13.02–15.84)
**(g) Tabulated by drainage system faults**								
No faults	84692	122	2.14 (2.04–2.24)	20	0.34 (0.30–0.37)	70095	143	3.33 (3.20–3.46)
Faults	2160	1811	5.65 (4.67–6.62)	284	0.93 (0.52–1.33)	1831	2334	7.81 (6.58–9.04)
**(h) Tabulated by problems in area**								
No problems	15813	241	1.52 (1.33–1.72)	53	0.34 (0.25–0.43)	14181	381	2.69 (2.42–2.95)
Slight problems	46454	907	1.95 (1.83–2.08)	124	0.27 (0.22–0.31)	39541	1235	3.12 (2.95–3.29)
Moderate problems	17259	491	2.84 (2.60–3.09)	75	0.43 (0.34–0.53)	13165	544	4.13 (3.79–4.47)
Substantial problems	8454	319	3.77 (3.37–4.18)	59	0.69 (0.52–0.88)	5994	364	6.07 (5.47–6.68)
**(i) Tabulated by tenure type**								
Owner occupied	45777	837	1.83 (1.71–1.95)	135	0.29 (0.25–0.34)	43584	1311	3.01 (2.85–3.17)
Private rented	11454	382	3.34 (3.01–3.66)	74	0.65 (0.50–0.79)	8478	370	4.36 (3.93–4.80)
Local authority	18824	503	2.67 (2.44–2.90)	54	0.29 (0.21–0.36)	12982	554	4.27 (3.92–4.61)
Housing association (RSL)	11966	237	1.98 (1.73–2.23)	48	0.40 (0.29–0.51)	7873	293	3.72 (3.30–4.14)
**(j) Tabulated by dwelling type**								
End terrace	9942	258	2.60 (2.28–2.91)	38	0.38 (0.26–0.50)	9757	309	3.17 (2.82–3.51)
Mid terrace	18321	553	3.02 (2.77–3.27)	88	0.48 (0.38–0.58)	18019	574	3.19 (2.93–3.44)
Semi detached	25752	398	1.55 (1.39–1.70)	64	0.25 (0.19–0.31)	25600	863	3.37 (3.15–3.59)
Detached	14813	376	2.54 (2.29–2.79)	66	0.45 (0.34–0.55)	14780	636	4.30 (3.98–4.63)
Purpose built flat	16141	265	1.64 (1.45–1.84)	39	0.24 (0.17–0.32)	3411	103	3.02 (2.45–3.59)
Other	3004	109	3.63 (2.96–4.30)	14	0.47 (0.22–0.71)	1333	42	3.15 (2.21–4.09)
**(k) Tabulated by date of construction**								
Pre 1850	2698	192	7.12 (6.15–8.09)	42	1.56 (1.09–2.02)	2387	238	9.97 (8.77–11.17)
1850–1899	7353	300	4.08 (3.63–4.53)	59	0.80 (0.60–1.01)	6237	264	4.23 (3.73–4.73)
1900–1918	7023	266	3.79 (3.34–4.23)	40	0.57 (0.39–0.75)	6345	278	4.38 (3.88–4.88)
1919–1944	15245	390	2.56 (2.31–2.81)	61	0.40 (0.30–0.50)	14276	559	3.92 (3.60–4.23)
1945–1964	20552	352	1.71 (1.54–1.89)	46	0.22 (0.16–0.29)	17611	532	3.02 (2.77–3.27)
1965–1980	20098	284	1.41 (1.25–1.58)	36	0.18 (0.12–0.24)	14691	377	2.57 (2.31–2.82)
Post 1980	15049	175	1.16 (0.99–1.33)	27	0.18 (0.11–0.25)	11369	280	2.46 (2.18–2.75)
**(l) Tabulated by region**								
East Midlands	7912	164	2.07 (1.76–2.39)	20	0.25 (0.14–0.36)	7085	271	3.82 (3.38–4.27)
Eastern	9137	153	1.67 (1.41–1.94)	26	0.28 (0.18–0.39)	7801	280	3.59 (3.18–4.00)
London	12177	587	4.82 (4.44–5.20)	65	0.53 (0.40–0.66)	7583	212	2.80 (2.42–3.17)
North East	6152	93	1.51 (1.21–1.82)	14	0.23 (0.11–0.35)	5440	145	2.67 (2.24–3.09)
North West & Merseyside	12041	204	1.69 (1.46–1.92)	40	0.33 (0.23–0.43)	10445	281	2.69 (2.38–3.00)
South East	12870	210	1.63 (1.41–1.85)	37	0.28 (0.19–0.38)	10781	334	3.10 (2.77–3.42)
South West	9271	144	1.55 (1.30–1.80)	37	0.40 (0.19–0.38)	8022	281	3.50 (3.10–3.90)
West Midlands	8598	164	1.91 (1.61–2.20)	45	0.52 (0.37–0.68)	7387	424	5.74 (5.21–6.27)
Yorkshire & Humberside	9863	240	2.43 (2.13–2.74)	27	0.27 (0.17–0.38)	8373	300	3.58 (3.19–3.98)
**(J) Tabulated by previous rodent control operations**								
No	1797	304	16.92 (15.19–18.65)	55	3.06 (2.26–3.86)	1591	559	35.14 (32.79–37.48)
Yes	4703	1531	32.55 (31.22–33.89)	239	5.08 (4.45–5.71)	4246	1336	31.47 (30.07–32.86)
**(H) Tabulated by month of survey**								
January	6272	167	2.66 (2.26–3.06)	29	0.46 (0.29–0.63)	5207	223	4.28 (3.73–4.83)
February	9024	220	2.44 (2.12–2.76)	30	0.33 (0.21–0.45)	7482	229	3.06 (2.67–3.45)
March	3226	88	2.73 (2.17–3.29)	11	0.34 (0.14–0.54)	2655	95	3.58 (2.87–4.28)
April	4169	102	2.45 (1.98–2.92)	18	0.43 (0.23–0.63)	3444	142	4.12 (3.46–4.79)
May	10078	212	2.10 (1.82–2.38)	36	0.36 (0.24–0.47)	8332	283	3.40 (3.01–3.79)
June	6735	139	2.06 (1.72–2.40)	25	0.37 (0.23–0.51)	5676	208	3.66 (3.18–4.15)
July	12285	259	2.11 (1.85–2.36)	49	0.40 (0.29–0.51)	10210	343	3.36 (3.01–3.71)
August	10307	196	1.90 (1.64–2.17)	30	0.29 (0.19–0.40)	8522	289	3.39 (3.01–3.78)
September	4151	72	1.73 (1.34–2.13)	8	0.19 (0.06–0.33)	3419	113	3.31 (2.71–3.90)
October	7781	162	2.08 (1.76–2.40)	33	0.42 (0.28–0.57)	6467	223	3.45 (3.00–3.89)
November	11733	284	2.42 (2.14–2.70)	36	0.31 (0.21–0.41)	9645	323	3.35 (2.99–3.71)
December	1809	47	2.60 (1.87–3.33)	4	0.22 (0.00–0.44)	1489	48	3.22 (2.33–4.12)

The presence of litter around the dwelling (OR: 1.83–2.18), keeping pets or livestock outside (OR: 1.30–2.77) and drainage system faults (OR: 1.73–2.10) all increased the odds of mice being present indoors and rats being present indoors and around the dwelling. An interaction between litter and region for rats inside was found, presence of litter appeared to be associated with disproportionately high levels of indoor rat occurrence in London, North West and Merseyside, South West and the West Midlands (these were also the four geographical regions with the highest odds of indoor rodent prevalence).

Compared to those in city centres, rural dwellings were more likely to have mice inside (OR: 3.06), more likely to have rats inside (OR: 5.63) and more likely to have rats around them (OR: 3.24). Odds of mice being present inside the dwelling (OR: 1.33) and rats being present around it (OR: 1.41) were highest for areas with the lowest density of housing; the number of dwellings in the local area did not affect the probability of reporting rats inside properties. The odds of indoor rat presence were lowest for post-1850 properties and gradually declined with dwelling age.

Properties on wider plots (>20 m wide) were more likely to have mice inside (OR: 1.55) or rats outside (OR: 1.50) than properties on plots with the same width as the dwelling. Properties on narrower plots (<10 m wide) were less likely to have rats inside than properties on plots with the same width as the dwelling (OR: 0.52). Dwellings situated in a cul-de-sac or crescent (with no through traffic) had lower prevalence of mice compared to major trunk roads; rats were less prevalent around dwellings situated on side roads and private roads compared to major trunk roads.

Rented properties were more likely to have mice inside than owner occupied properties (OR: 1.31–1.84); housing association properties were more likely to have rats inside than owner occupied properties (OR: 1.92) but rented dwellings occupied under other tenure types were not. Rats were more prevalent around local authority (OR: 1.50) and housing association-owned rented properties (OR: 1.32) but not privately-owned rental properties.

Purpose built flats (OR: 0.72) and semi-detached houses (OR: 0.73) had lower odds of reporting mice inside than end terrace properties, although in London, prevalence of mice was disproportionately higher in end terraces and mid-terraces (Figure S2). The odds of reporting mice inside (OR: 1.28–1.82) and rats outside (OR: 1.35–2.65) were higher in areas with slight, moderate or substantial problems; odds of reporting rats inside were higher in areas with substantial problems (OR: 1.97). Mice were more prevalent in London compared to East Midlands (OR: 3.08); they were less prevalent in the South West (OR: 0.70). Rats were more prevalent inside (OR: 2.01) and around (OR: 1.77) dwellings in the West Midlands and also more prevalent inside dwellings in London (OR: 2.69).

## Discussion

We examined housing survey data collected during the period 1996–2010 to determine the prevalence of commensal rodents in and around domestic dwellings in England and then explored factors associated with prevalence. We used weighted and un-weighted data to calculate multi-year rodent prevalence estimates for consistency with other EHCS and EHS national housing statistics, but thereafter we used un-weighted data for consistency across survey periods, with the repeated records removed. It should be noted therefore that the results of the predictive models apply to the data sample that we used, and caution should be exercised if extrapolating prevalence estimates for different dwelling categories to national totals, although the relatively large data set available to us means that it is more likely that the data are representative.

Weighted and un-weighted rodent prevalence varied over time and followed similar trends; there were two peaks in prevalence of mice inside dwellings and rats around dwellings, the first in 2002 and the second in 2008 which we modelled using un-weighted data. The time lag between peaks in prevalence was similar to the well-documented regular oscillations of northern European microtine rodent populations, where population cycles of 4–6 years are not uncommon^26,27^. Our data collection has been discontinued and hence we do not know what the trends are post-2010. Although we could not include variables on rodent control activity in our models (because the data were usually only collected for dwellings with a current rodent problem), there is likely to be a close link between rodent prevalence and rodent control activities^28,29^; data collected by pest control providers on the number of treatments carried out each year could potentially be used as a proxy for rodent prevalence. If the peaks in prevalence observed in our data were a result of regular variations in population size, we would expect a peak in rodent control treatments in or around 2014 and the next in 2020. Data from local authorities indicated a peak in rodent control treatments in 2012–2013^30^, which fits reasonably well with our predictions, although data that also included rodent control treatments carried out by commercial companies (and ideally householders) would likely provide a better predictor.

It has previously been suggested that reductions in pest control provision by local authorities could result in increases in rodent problems. The National Pest Technicians Association for example reported that the number of rodent control treatments carried out by local authorities in the UK declined by nearly 50% between 2005/6 and 2010/11, and linked this to increased levels of charging for domestic rodent control treatments in the public sector, and potentially, as a consequence, a greater level of householder tolerance towards rodent presence^31^. However, it does not necessarily follow that the overall level of rodent control effort (including private or commercial pest control provision) has declined nationally and we found no evidence that rodent control effort had consistently declined during 1996–2010; the trends matched those seen in rodent prevalence with a peak in 2008 for the proportion of dwellings where action against rodents had been or was currently being taken (Figure S6). Why the proportion of dwellings taking action increases at times of increased rodent prevalence is not immediately obvious; logically a householder should be equally likely to respond to the presence of rodents regardless of the national trend in prevalence. However, prevalence is likely to be linked to abundance, hence peaks in prevalence could be associated with peaks in abundance (i.e. a greater number of rodents per dwelling) that householders would be less likely to ignore or overlook. The issue of commensal rodents in rented properties was highlighted recently in England following a poll of private tenants where 11.3% had problems with vermin over a single year, although these figures included ants and cockroaches as well as rodents^32^. We found no evidence that likelihood of taking action against a current rodent problem was lower for rented dwellings, despite reports of difficulties in establishing whether tenants or landlords are responsible^24^. Our data did however confirm that some categories of rodent prevalence were higher for rented dwellings compared to those under owner occupancy.

Other dwelling-specific variables consistently associated with increased rodent prevalence were litter in the immediate vicinity of the dwelling, pets and/or livestock in the garden and drainage faults. Litter provides potential harbourage (protection from predators) and nest sites for rodents; in Salzburg (Austria) for example, Norway rat occurrence was lower in urban areas with no vegetation^33,34^. High levels of rodent harbourage probably also makes early detection and treatment of rodent problems more difficult. Pets or livestock in the garden have previously been linked with greater levels of rodent prevalence in England^11^ probably as a result of access for rats to animal feed, increased availability of harbourage from bedding stored in the vicinity or both. Similarly, in Madrid, Norway rats were more frequently reported near cat feeding stations^35^. It is recommended therefore that householders keeping pets or livestock in their gardens take all possible steps to deter commensal rodents by storing animal feed and bedding in rodent-proof conditions, and using rodent-proof animal feeders where possible. Drainage faults have also previously been linked to increased rodent prevalence in England^11^, and in Budapest, rats were most frequently encountered in sewer inspection points^29^. It is still unclear however why drainage faults might lead to increased prevalence of mice in dwellings and we could find no references to house mice living in sewer systems; although they sometimes excavate burrows, house mice are more typically associated with dry habitats^36^. It is possible that drainage faults are linked with other property defects not included in our study; similarly, date of construction could be linked to other property defects that could facilitate rodent access, or other factors not included in the model such as presence of unoccupied cellars which has previously been linked with increased Norway rat occurrence^29^. Date of dwelling construction has previously been linked with occurrence of Norway rats in England^11^ and Madrid^35^.

Variables relating to the local area in which the dwelling was located were also associated with rodent prevalence; rural areas and lowest levels of housing density were linked with higher prevalence of rats outdoors and house mice indoors. Urban-rural gradients in rodent distribution have previously been reported with some species (vesper mice *Calomys* sp.) favouring rural habitats, others (ship rats *Rattus rattus*) favouring highly urbanised areas, and Norway rats and house mice intermediate^10^. Farmland house mouse populations have declined in Britain with modernisation of farming practices^37^ but populations persist in and around farm buildings; dispersal between populations is low^38,39^ although higher rates of dispersal for rural populations have been reported for house mice and other rodents compared to their urban counterparts^37,40^. Increased isolation potentially makes urban rodent populations easier to manage than rural populations as re-invasion following control measures is reduced, offering a potential explanation for the lower rodent prevalence in urban areas seen here. In a previous study, regional differences were found for prevalence of mice inside and rats outside dwellings^11^; similar patterns of regional differences were found in the present analyses and the West Midlands was again found to have the highest prevalence of rats outside, although London dwellings had the highest prevalence of mice instead of Yorkshire/Humberside. A significant interaction between dwelling type and region indicated that end terrace and mid terrace dwellings in London had disproportionately higher prevalence of mice compared to other regions. The reasons for this are unclear, although dwelling size, which was not included in our models may be involved. We also found regional differences in prevalence of rats inside dwellings, with highest prevalence in London and the West Midlands. There appeared to be interesting regional differences in the effect of litter around the dwelling which may be driving regional differences in prevalence of rats indoors; in some regions, notably those with higher prevalence of rats indoors, presence of litter appeared to disproportionately increase prevalence of rats compared to other regions. Why presence of litter in some regions is more likely to result in presence of rats indoors is unclear, but targeted litter prevention schemes in these regions would likely be of more benefit than in others.

Overall model fit for each of the three categories of rodent prevalence was fair; with AUC midway between chance (0.5) and perfect (1.0) model fit. This suggests that there are other drivers of rodent prevalence which were not included in our models; over-feeding of wild birds and poor management of domestic waste have previously been linked with increased rodent problems^41^; close proximity to watercourses and higher levels of human population density have also been linked with increased occurrence of rats^8,33^ (we included housing density in our models but not human population density). Other potential drivers include any activities that potentially provide resources to attract and sustain rodent populations. Density-dependent population processes and climatic changes can also influence rodent populations^42,43^; climatic factors were linked with reports of urban Norway rats in Madrid, although reporting bias (increased likelihood of reporting during times of increased outdoor activity by residents) potentially confounded these effects^44^. For consistency with previous analyses^11^, we determined rodent presence from either physical evidence or householder response, although due to the systematic nature of our surveys it is less likely that reporting bias could explain the changes in prevalence apparent in our data. Increased occurrence of Norway rats in British farm buildings during autumn and winter has been reported^45^ and is probably a response to changes in distribution of food resources and climatic factors, these are likely to vary to a lesser extent in urban habitats and we found no significant evidence for seasonal shifts in occurrence of Norway rats in dwellings, although models including month of survey provided a similar fit for the data and further data exploration could potentially reveal urban-rural differences. We did however find that prevalence of mice was lower in summer than in spring or autumn as has previously been reported^46^.

In summary, we found evidence for two peaks in rodent prevalence in or around domestic dwellings in England during the period 2001–2010. Several risk factors were associated with rodent prevalence including litter around the dwelling, pets and/or livestock in the garden, drainage faults, housing density, the urban-rural nature of the area and tenure type. It is likely that these household variables are drivers of commensal rodent abundance, however identifying predictive relationships between ecological mechanisms and population growth rates is challenging through observation of natural populations alone^47^, and further work should seek to explore the possible drivers of commensal rodent populations by experimental manipulation.

## Electronic supplementary material

Supplementary Information
