# Supplementary material for: Results of a 15-year systematic survey of commensal rodents in English dwellings
Source: Sci Rep. 2017 Nov 21;7:15882. doi: 10.1038/s41598-017-15723-9 (PMC5698335; doi:10.1038/s41598-017-15723-9)
Supplement: Supplementary file 1 — Supplementary Information [file 41598_2017_15723_MOESM1_ESM.pdf]

Results of a 15-year systematic survey of two common commensal rodents in England

Lambert, M., Vial, F., Pietravalle, S., Cowan, D.

Supplementary Method: Description of variables used in the analyses - adapted from the *English Housing Survey Surveyor Briefing Manual; Completing the Survey Form* (March 2009).

Litter;

The level of litter and rubbish around the dwelling was assessed by surveyors in order to provide an indication of household attitudes to home and its maintenance, as well providing an indication of the levels of harbourage for rats and mice and other health hazards. The assessment was applied to private gardens and plots and did not include well managed compost heaps. More recently, a simple (3-category) scoring system has been used to assess the level of litter or rubbish, but this information was not available for the majority of surveys, instead presence or absence of litter and rubbish was used.

Plot width;

Plot width in metres was measured (to the nearest metre) from left hand to right hand edge.

Pets/livestock kept outside

Recorded presence or absence of rabbit hutches, chicken runs, aviaries, dog kennels, etc. outside (including in garages, sheds and stables) which might attract vermin..

Situation of block;

Concerns the nature of the road on which the dwelling is located. This is an indication of the amount of through traffic and its consequence for noise. If a block was set back from a main road, either by grass planting or small slip roads or both, the situation was described as that of the main road.

- Major trunk road – a dual carriageway or very busy ‘A’ road.
- Main road – A road linking different parts or suburbs or a town or city, or linking villages.
- Side road – typically a road off a main road which is not a cul-de-sac or crescent.
- Cul-de-sac/crescent – road with no through traffic
- Private road – un-adopted road with access only for residents or approved persons

- Unmade/no road – typically a track to a farmhouse or remote cottage.

Drainage system faults;

Survey data for presence or absence of faults in underground drainage including blockages or other faults relevant to Health and Safety risks were aggregated into a single yes/no response.

Tenure type;

Tenures relates to the ownership of the dwelling.

- Owner occupied dwellings includes properties owned outright or under a mortgage agreement, and those in shared ownership and part ownership with a local authority or housing association, and dwellings bought from a local authority.

- Private rented includes dwellings rented from a private landlord, private company, other organisation, relative or friend and includes properties owned by Defence Estates (MOD) but not managed by Housing Associations.

- Local authority includes properties rented from the local authority or any new town or development Corporation.

- Housing association includes properties rented from a housing association, including co-operatives and housing charitable trusts and includes properties owned by Defence Estates (MOD) but managed by Housing Associations.

Dwelling type;

- End terrace – the end of a row of more than 2 attached properties

- Mid terrace – a dwelling attached on both sides

- Semi-detached – code only if dwelling is one of a pair.

- Detached – including link-detached dwellings.

- Temporary – e.g. caravan or houseboat.

- Purpose-built flats – building was originally built containing flats. Residential accommodation over shops and other non-residential included if the flats have their own separate access.

- Other - converted flats (buildings converted into individual flats which have been defined as separate dwellings and non-residential plus flat where residents must pass through non-residential accommodation to gain access to their living accommodation.

Date of construction;

Date of original construction. If a property had a large later extension or had been partially rebuilt, age of the oldest part was recorded even if it accounted for less than half of the area of dwelling.

Region;

Location of dwelling by Government Office Region (GOR).

Variables relating to the LOCAL AREA.

Surveyors were asked to define the ‘area around the dwelling of which the dwelling seems to be a part’ and estimate the number of dwellings in that area. This Local Area was likely to be, but was not necessarily, defined by physical boundaries such as roads, railway lines, canals, etc. The survey dwelling was not necessary at the centre of the area; surveyors were asked to define an area of manageable size so that they could clearly define the boundaries of the local area and visually inspect the whole area on foot before proceeding to complete the following questions:

Number of dwellings in area;

- 500+
- 300-499
- 100-299
- 50-99
- 25-49
- Less than 25

Nature of area;

- Commercial City/Town Centre – this is the area that would constitute part/all of the centre of a city or town. Areas do not have to be run down to be coded as city or town centre. It is likely that these areas will have a high percentage of commercial properties such as shops and businesses.

- Urban – this is the area around the core of towns and cities, and also older urban areas which have been swallowed up by a metropolis. Areas would be largely but not exclusively residential.

- Suburban residential – this is the outer area of towns or cities, and would include large, planned housing estates on the outskirts of towns or larger areas of older residential stock.

- Rural residential – these can be free standing residential areas or suburban areas of villages, often meeting the housing needs of people who work in nearby towns and cities.

- Village centre – these are traditional English villages or the old heart of villages which have been suburbanised.

- Rural – these areas are predominantly rural e.g. agricultural with isolated dwellings or small hamlets.

Problems in area;

Depending on the year of the survey, up to 16 categories of problems in the local area, such as litter, neglected buildings, scruffy gardens, vacant buildings and vandalism, were scored by the surveyors on a scale of 1 (no problems) to 5 (major problems). The mean score from 14 of these categories ($\bar{x}$) was used to allocate dwellings to one of four categories; no problems ($\bar{x} = 1$), slight problems ($1 < \bar{x} < 1.5$), moderate problems ($1.5 < \bar{x} < 2.0$) or substantial problems ($\bar{x} > 2.0$). This variable was treated as a factor in the models; the 14 categories (selected because they were recorded for all survey years) were vacant sites, intrusive industry, non-conforming uses, vacant boarded-up buildings, ambient air quality, heavy traffic, intrusion from motorways and arterial roads, noise from railways or aircraft, nuisance from on-street parking, litter and rubbish, scruffy gardens, vandalism, graffiti, and scruffy or neglected buildings.

Rodent control;

Where the householder reported a current problem the surveyor asked to see evidence and asked two additional questions;

- Has anyone treated the rats/mice problem?

- Is anything currently being done to stop or control the rats/mice problem?

If the answer to the first question was yes, further details were recorded on how the problem was treated and by whom.

Supplementary Table S1: Number (n) and percentage of occupied dwellings with mice present.

Year	Mice inside			
	**Sample**	**n**	**Weighted %**	**Un-weighted %**
2009 - 2010	16047	373	2.24 (1.98 - 2.50)	2.32 (2.09-2.56)
2008 - 2009	15512	411	2.46 (2.20 - 2.73)	2.65 (2.40-2.90)
2007 - 2008	15523	409	2.46 (2.19 - 2.73)	2.63 (2.38-2.89)
2006 - 2007	15604	358	2.07 (1.82 - 2.32)	2.29 (2.06-2.53)
2005 - 2006	15648	339	1.94 (1.70 - 2.17)	2.17 (1.94-2.39)
2004 - 2005	16059	345	2.04 (1.80 - 2.28)	2.15 (1.92-2.37)
2003 - 2004	15874	344	2.02 (1.79 - 2.26)	2.17 (1.94-2.39)
2002 - 2003	15950	381	2.14 (1.90 - 2.38)	2.39 (2.15-2.63)
2001	16721	285	1.44 (1.21 - 1.61)	1.70 (1.51-1.90)
1996	11691	240	1.84 (1.53 – 2.15)	2.05 (1.80-2.31)

Supplementary Table S2: Number (n) and percentage of occupied dwellings with rats present.

Year	Rats inside				Rats outside			
	Sample	n	Weighted %	Un-weighted %	Sample	n	Weighted %	Un-weighted %
2009 - 2010	16047	53	0.29 (0.20 - 0.39)	0.33 (0.24-0.42)	13375	444	3.11 (2.80 - 3.42)	3.32 (3.02-3.62)
2008 - 2009	15512	59	0.34 (0.24 - 0.44)	0.38 (0.28-0.48)	12860	505	3.64 (3.29 - 3.99)	3.92 (3.59-4.26)
2007 - 2008	15523	71	0.42 (0.31 - 0.54)	0.46 (0.35-0.56)	12786	494	3.46 (3.12 - 3.80)	3.86 (3.53-4.26)
2006 - 2007	15604	57	0.37 (0.26 - 0.48)	0.37 (0.27-0.46)	12976	455	3.04 (2.72 - 3.36)	3.51 (3.19-3.82)
2005 - 2006	15648	60	0.36 (0.25 - 0.46)	0.38 (0.29-0.48)	13062	444	3.10 (2.78 - 3.42)	3.40 (3.09-3.71)
2004 - 2005	16059	56	0.30 (0.21 - 0.39)	0.35 (0.26-0.44)	13387	444	3.10 (2.79 - 3.42)	3.32 (3.01-3.62)
2003 - 2004	15874	46	0.24 (0.16 - 0.32)	0.29 (0.21-0.37)	13202	450	3.04 (2.73 - 3.34)	3.41 (3.10-3.72)
2002 - 2003	15950	58	0.31 (0.22 - 0.40)	0.36 (0.27-0.46)	13090	494	3.24 (2.92 - 3.56)	3.77 (3.45-4.10)
2001	16721	47	0.27 (0.18 - 0.36)	0.28 (0.20-0.36)	13805	432	2.94 (2.60 - 3.29)	3.13 (2.84-3.42)
1996	11691	36	0.35 (0.19 - 0.51)	0.31 (0.21-0.41)	9491	198	1.72 (1.33 - 2.12)	2.09 (1.80-2.37)

Supplementary Table S3: Number and percentage of occupied dwelling with rodents present by factor type. Percentages were weighted using the survey dwelling weights to derive nationally-representative estimates of occurrence (1996 – 2010).

	Sample	Mice inside		Rats inside		Rats outside		
		**n**	**Weighted %**	**n**	**Weighted %**	**Sample**	**n**	**Weighted %**
**a) Tabulated by litter**								
No	74697	1246	1.58 (1.48 - 1.69)	190	0.25 (0.21 - 0.30)	63161	1602	2.30 (2.16 - 2.44)
Yes	23171	927	3.90 (3.59 - 4.21)	153	0.64 (0.52 - 0.75)	17808	1106	5.85 (5.45 - 6.25)
**b) Tabulated by number of dwellings in area**								
500+	10208	279	2.52 (2.14 - 2.89)	32	0.30 (0.17 - 0.44)	7709	262	3.14 (2.69 - 3.58)
300-499	10669	213	1.81 (1.53 - 2.10)	34	0.33 (0.20 - 0.46)	8346	273	2.77 (2.39 - 3.16)
100-299	31854	606	1.74 (1.57 - 1.92)	81	0.21 (0.16 - 0.26)	25649	612	2.04 (1.85 - 2.23)
50-99	21994	381	1.46 (1.29 - 1.63)	69	0.29 (0.20 - 0.37)	18467	498	2.40 (2.12 - 2.67)
25-49	14184	250	1.68 (1.42 - 1.95)	43	0.31 (0.20 - 0.42)	12325	405	2.98 (2.64 - 3.31)
Less than 25	10623	467	4.10 (3.68 - 4.51)	86	0.72 (0.54 - 0.91)	9770	670	5.85 (5.35 - 5.35)
**c) Tabulated by width of plot**								
Same as dwelling	31827	842	2.53 (2.32 - 2.74)	141	0.44 (0.35 - 0.53)	29669	878	2.64 (2.44 - 2.85)
Less than 10m	23126	396	1.42 (1.24 - 1.60)	56	0.21 (0.14 - 0.27)	23126	691	2.40 (2.19 - 2.62)
Less than 20m	22809	382	1.45 (1.28 - 1.62)	56	0.24 (0.16 - 0.31)	22809	702	2.68 (2.44 - 2.93)
20m or more	5639	300	4.87 (4.25 - 5.5)	52	0.83 (0.58 - 1.08)	5637	407	6.77 (6.04 - 7.51)
**d) Tabulated by keeping pets outside**								
No	93505	1934	1.92 (1.82 - 2.03)	290	0.29 (0.25 - 0.34)	76407	2094	2.44 (2.31 - 2.57)
Yes	5578	244	3.80 (3.23 - 4.37)	53	0.90 (0.59 - 1.21)	5466	588	10.04 (9.1 - 10.98)
**e) Tabulated by road type**								
Major trunk road or main road	13460	358	2.55 (2.24 - 2.85)	68	0.51 (0.36 - 0.66)	10099	489	4.61 (4.15 - 5.06)
Side road	43954	1130	2.35 (2.18 - 2.52)	157	0.33 (0.27 - 0.39)	37019	1160	2.73 (2.55 - 2.91)

Cul de sac/crescent	37727	521	1.23 (1.1 - 1.36)	85	0.18 (0.14 - 0.23)	31776	852	2.24 (2.05 - 2.44)
Private road	3091	110	3.30 (2.54 - 4.07)	26	0.90 (0.45 - 1.34)	2209	117	4.55 (3.60 - 5.50)
Unmade/no road	1398	79	5.62 (4.18 - 7.06)	11	1.15 (0.33 - 1.98)	1241	104	9.01 (7.09 - 10.94)
**f) Tabulated by nature of area**								
City centre	3205	84	2.27 (1.63 - 2.91)	11	0.41 (0.09 - 0.74)	1455	55	2.95 (2.05 - 3.86)
Urban	21314	689	3.00 (2.74 - 3.26)	99	0.48 (0.36 - 0.59)	15161	506	2.92 (2.63 - 3.22)
Suburban residential	57436	868	1.29 (1.18 - 1.41)	127	0.19 (0.15 - 0.22)	49087	1201	2.07 (1.93 - 2.22)
Rural residential	11296	241	2.13 (1.82 - 2.45)	41	0.34 (0.22 - 0.46)	10609	417	3.59 (3.18 - 3.99)
Village centre	3652	103	2.70 (2.11 - 3.29)	20	0.50 (0.24 - 0.75)	3364	182	5.41 (4.56 - 6.26)
Rural	2715	243	8.58 (7.39 - 9.78)	49	1.84 (1.19 - 2.50)	2654	361	12.35 (10.98 - 13.72)
**g) Tabulated by drainage system faults**								
No faults	95500	2019	1.95 (1.84 - 2.05)	315	0.31 (0.27 - 0.36)	78882	2508	2.82 (2.69 - 2.96)
Faults	2489	138	5.68 (4.53 - 6.83)	23	0.92 (0.49 - 1.35)	2099	158	7.28 (5.96 - 8.59)
**h) Tabulated by problems in area**								
No problems	18223	283	1.49 (1.29 - 1.68)	59	0.27 (0.19 - 0.36)	16263	402	2.14 (1.90 - 2.37)
Slight problems	51422	987	1.82 (1.68 - 1.96)	136	0.27 (0.21 - 0.32)	43675	1311	2.77 (2.59 - 2.95)
Moderate problems	20024	561	2.69 (2.42 - 2.97)	83	0.41 (0.30 - 0.51)	15318	603	3.55 (3.22 - 3.89)
Substantial problems	9811	364	3.32 (2.91 - 3.72)	69	0.69 (0.47 - 0.90)	6954	404	5.27 (4.66 - 5.89)
**i) Tabulated by tenure type**								
Owner occupied	51670	958	1.76 (1.64 - 1.89)	156	0.29 (0.23 - 0.34)	49212	1424	2.68 (2.53 - 2.84)
Private rented	12260	410	3.09 (2.74 - 3.45)	81	0.63 (0.46 - 0.79)	9119	388	4.00 (3.53 - 4.46)
Local authority	22227	574	2.57 (2.32 - 2.81)	60	0.27 (0.19 - 0.35)	15248	594	3.63 (3.31 - 3.96)
Housing	13555	257	2.12 (1.81 - 2.42)	50	0.38 (0.26 - 0.49)	8830	320	3.48 (3.05 - 3.91)

association (RSL)								
**j) Tabulated by dwelling type**								
End terrace	11347	283	2.29 (1.97 - 2.61)	45	0.37 (0.24 - 0.50)	11131	345	2.73 (2.39 - 3.08)
Mid terrace	21168	618	2.72 (2.45 - 2.98)	97	0.45 (0.34 - 0.56)	20805	637	2.65 (2.40 - 2.89)
Semi detached	28986	471	1.41 (1.26 - 1.57)	70	0.21 (0.15 - 0.26)	28815	949	2.69 (2.49 - 2.90)
Detached	16072	421	2.25 (2.02 - 2.49)	73	0.39 (0.29 - 0.49)	16032	669	3.71 (3.38 - 4.04)
Purpose built flat	18645	281	1.54 (1.29 - 1.80)	43	0.24 (0.13 - 0.35)	4049	107	2.24 (1.73 - 2.75)
Other	3442	125	3.28 (2.53 - 4.02)	17	0.51 (0.16 - 0.86)	1557	48	2.72 (1.71 - 3.73)
**k) Tabulated by date of construction**								
Pre 1850	3099	221	7.29 (6.20 - 8.38)	48	1.39 (0.91 - 1.87)	2758	258	8.78 (7.61 - 9.95)
1850-1899	8834	349	3.46 (3.03 - 3.89)	67	0.68 (0.49 - 0.88)	7542	305	3.64 (3.18 - 4.11)
1900-1918	8221	293	3.37 (2.92 - 3.82)	47	0.56 (0.38 - 0.75)	7446	310	3.95 (3.45 - 4.45)
1919-1944	17834	454	2.29 (2.04 - 2.54)	69	0.38 (0.28 - 0.49)	16701	616	3.28 (2.98 - 3.58)
1945-1964	23309	402	1.50 (1.32 - 1.69)	49	0.18 (0.12 - 0.24)	19778	561	2.42 (2.18 - 2.67)
1965-1980	22305	303	1.24 (1.07 - 1.42)	38	0.12 (0.07 - 0.16)	16124	388	2.06 (1.81 - 2.31)
Post 1980	16093	177	0.97 (0.79 - 1.16)	29	0.21 (0.11 - 0.31)	12047	288	2.03 (1.71 - 2.35)
**l) Tabulated by region**								
East Midlands	9147	185	1.91 (1.58 - 2.24)	21	0.28 (0.14 - 0.43)	8162	289	3.28 (2.85 - 3.71)
Eastern	10161	164	1.64 (1.34 - 1.95)	32	0.35 (0.21 - 0.49)	8656	302	3.10 (2.71 - 3.49)
London	13968	621	3.99 (3.60 - 4.38)	71	0.47 (0.33 - 0.60)	8759	228	2.19 (1.87 - 2.52)
North East	7340	116	1.48 (1.14 - 1.83)	21	0.23 (0.12 - 0.34)	6515	167	2.31 (1.89 - 2.73)
North West & Merseyside	13728	240	1.50 (1.26 - 1.73)	44	0.26 (0.17 - 0.36)	11851	314	2.09 (1.81 - 2.37)
South East	13852	225	1.62 (1.37 - 1.86)	38	0.26 (0.17 - 0.35)	11591	353	2.83 (2.44 - 3.22)
South West	10355	167	1.49 (1.23 - 1.75)	38	0.35 (0.23 - 0.48)	8937	291	2.94 (2.56 - 3.32)
West Midlands	9961	199	1.92 (1.60 - 2.24)	49	0.48 (0.30 - 0.66)	8499	463	4.73 (4.23 - 5.22)
Yorkshire &	11200	282	2.10 (1.80 - 2.40)	33	0.22 (0.14 - 0.31)	9439	319	2.98 (2.58 - 3.38)

Humberside

m) Tabulated by month of survey

January	6272	167	2.48 (2.06-2.89)	29	0.42 (0.26-0.59)	5207	223	3.86 (3.30-4.42)
February	9024	220	2.35 (1.97-2.74)	30	0.33 (0.20-0.47)	7482	229	2.76 (2.36-3.16)
March	3230	88	2.76 (2.03-3.47)	11	0.25 (0.09-0.41)	2659	95	3.13 (2.43-3.82)
April	4171	102	2.31 (1.79-2.83)	18	0.36 (0.18-0.54)	3446	142	3.67 (2.99-4.35)
May	10087	212	1.83 (1.53-2.12)	36	0.30 (0.19-0.42)	8340	283	2.87 (2.48-3.26)
June	6735	139	1.95 (1.57-2.33)	25	0.43 (0.23-0.64)	5676	208	3.38 (2.84-3.92)
July	12286	259	1.87 (1.61-2.14)	49	0.37 (0.25-0.48)	10211	343	3.00 (2.64-3.36)
August	10312	196	1.70 (1.42-1.98)	30	0.28 (0.156-0.41)	8527	289	3.12 (2.68-3.55)
September	4151	72	1.71 (1.21-2.20)	8	0.16 (0.04-0.28)	3419	113	3.01 (2.37-3.66)
October	7785	162	1.82 (1.50-2.14)	34	0.41 (0.26-0.56)	6471	223	3.01 (2.62-3.52)
November	11739	284	2.33 (2.00-2.66)	36	0.29 (0.17-0.41)	9651	323	3.13 (2.74-3.52)
December	1810	47	2.22 (1.50-2.93)	4	0.19 (0.00-0.39)	1490	48	2.72 (1.87-3.56)

Supplementary Table S4: Exploration of temporal patterns in the prevalence of mice inside dwellings. The best model (highlighted in purple) was the model with the lowest AIC and a significant reduction in model deviance (Chi-square test for analysis of deviance) compared to the null model.

Model	AIC	Analysis of deviance (p-value)
S(Year)+S(Month)	18647.14	<0.001
Year (factor)+S(Month)	18651.56	<0.001
S(Year)	18651.92	<0.001
Year only (factor)	18655.64	<0.001
S(Year)+Month (factor)	18657.55	<0.001
Year+S(Month)	18658.51	<0.001
Year (factor) + Month (factor)	18661.99	<0.001
S(Month)	18664.8	<0.001
Year only	18668.32	<0.001
Year + Month (factor)	18668.8	<0.001
Month only (factor)	18675.34	0.004
Null model	18681.1	

Supplementary Table S5: Exploration of temporal patterns in the prevalence of rats inside dwellings. The best model (highlighted in purple) was the most parsimonious model with the lowest AIC. ΔAIC < 2 are not deemed significant.[1] Models with the grey diagonal shading are thus not deemed more valid than the null model. For each model, the change in model deviance (Chi-square test for analysis of deviance) compared to the null model is also presented.

Model	AIC	Analysis of deviance (p-value)
Year only	4108.48	0.14
S(Year)	4108.49	0.14
Null model	4108.66	
S(Month)	4108.74	0.17
Year (factor)+S(Month)	4108.91	0.07
Year+S(Month)	4108.93	0.15
S(Year)+S(Month)	4108.94	0.15
Year only (factor)	4110.62	0.07
Year + Month (factor)	4117.86	0.25
S(Year)+Month (factor)	4117.87	0.25
Month only (factor)	4119.48	0.43
Year (factor) + Month (factor)	4121.65	0.14

Supplementary Table S6: Exploration of temporal patterns in the prevalence of rats outside dwellings. The best model (highlighted in purple) was the most parsimonious model with the lowest AIC. ΔAIC < 2 are not deemed significant.[1] Models with the grey diagonal shading are thus not deemed more valid than the model with only a smoother for year. For each model, the change in model deviance (Chi-square test for analysis of deviance) compared to the null model is also presented.

**Model**	**AIC**	**Analysis of deviance (p-value)**
S(Year)	21868.39	<0.001
S(Year)+S(Month)	21868.9	<0.001
S(Year)+Month (factor)	21869.14	<0.001
Year only (factor)	21873.51	0.002
Year (factor)+S(Month)	21874.14	0.003
Year (factor) + Month (factor)	21874.22	<0.001
S(Month)	21880.37	0.1
Null model	21881.13	
Year+S(Month)	21882.04	0.21
Year only	21882.63	0.47
Month only (factor)	21883.14	0.05
Year + Month (factor)	21884.67	0.06

Supplementary Table S7: Variance inflation factor (VIF) values were used to detect collinearity between the candidate explanatory variables. All VIF values were below 3, indicating there is no collinearity between variables, and hence no variables were excluded from the analysis[2]. VIF was calculated using the AED package in R version 3.2.4 .

Candidate Variable	GVIF
Litter	1.07
Number of dwellings in area	1.16
Width of plot	1.17
Animals outside	1.05
Road type	1.08
Nature of area	1.15
Drainage system faults	1.01
Problems in area	1.00
Tenure type	1.10
Dwelling type	1.06
Date of construction	1.17
Region	1.01
Month	1.02
Year	1.02

Supplementary Figure S1: receiver operating characteristic (ROC) curves for the three models

a) Mice inside dwellings

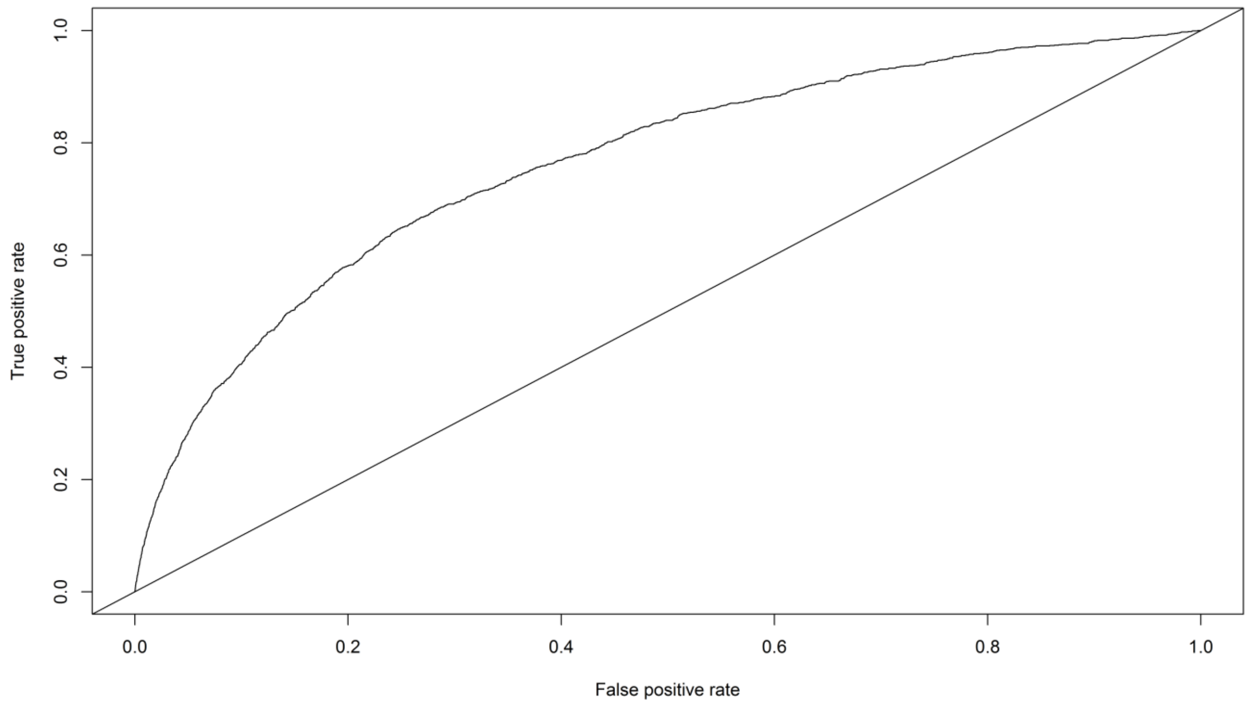

b) Rats inside dwellings

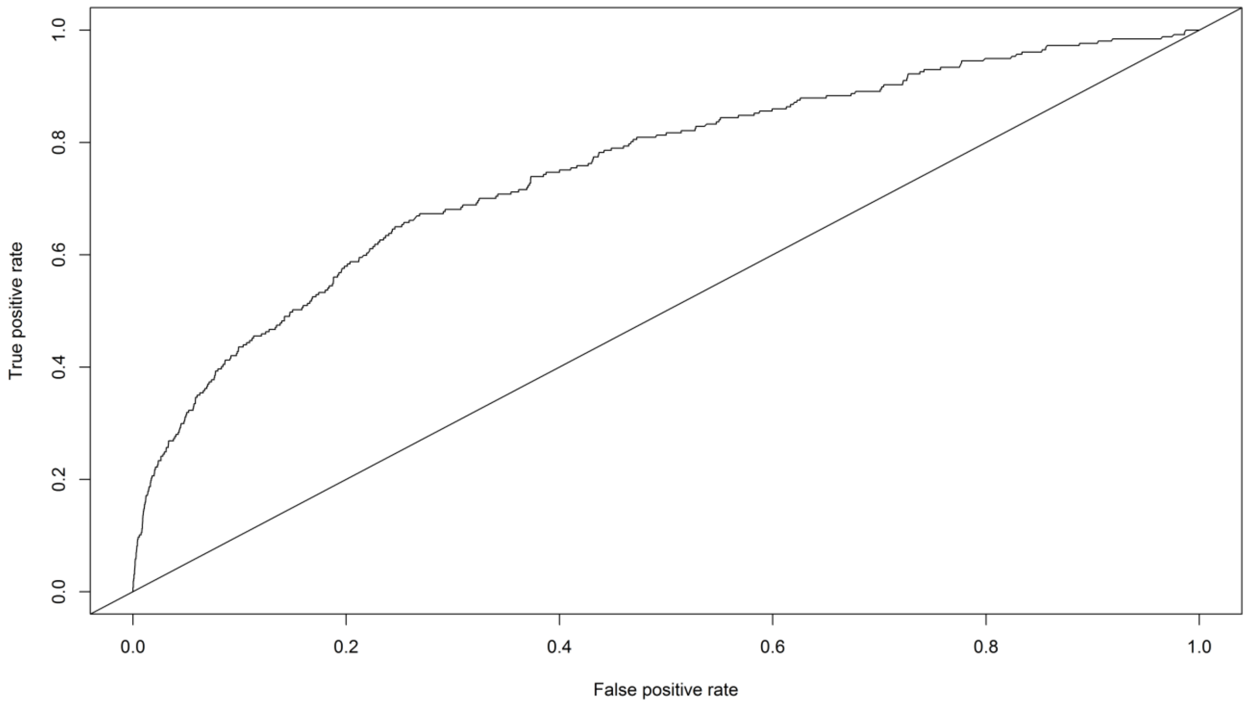

c) Rats outside dwellings

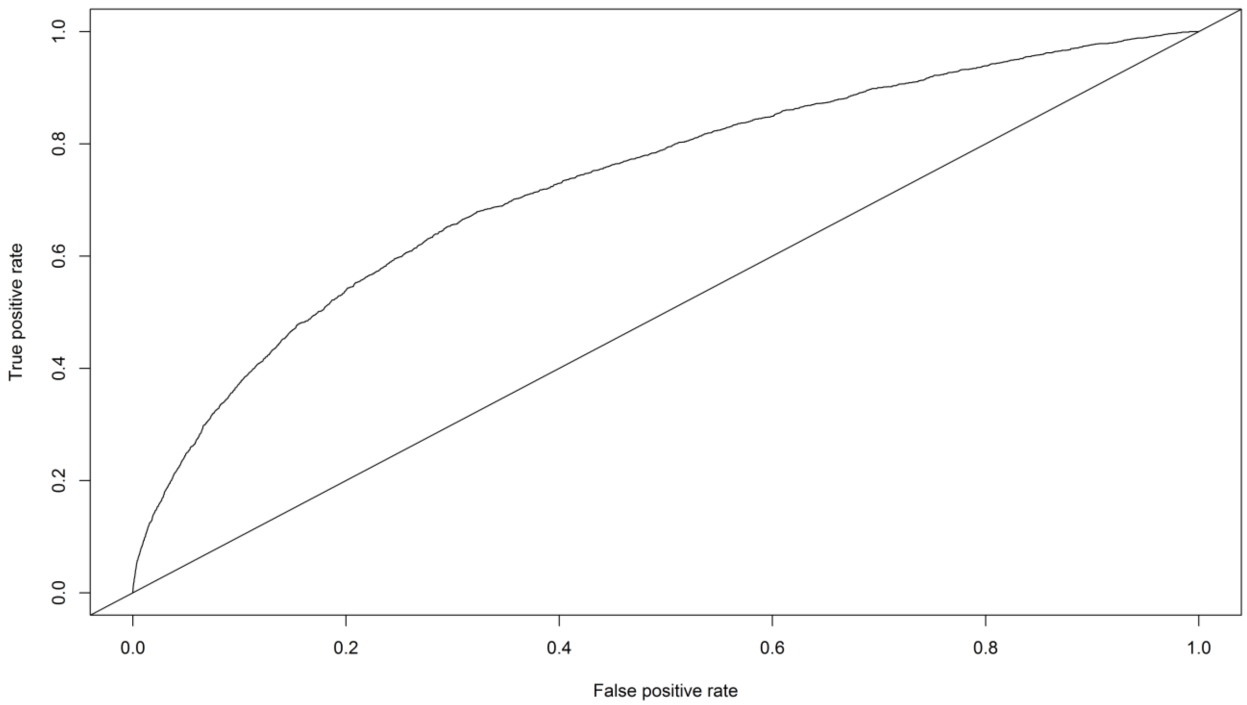

Supplementary Figure S2: Statistical significant (p<0.05) interactions between the variable region and other model variables on the prevalence of mice inside dwellings

a) Dwelling type

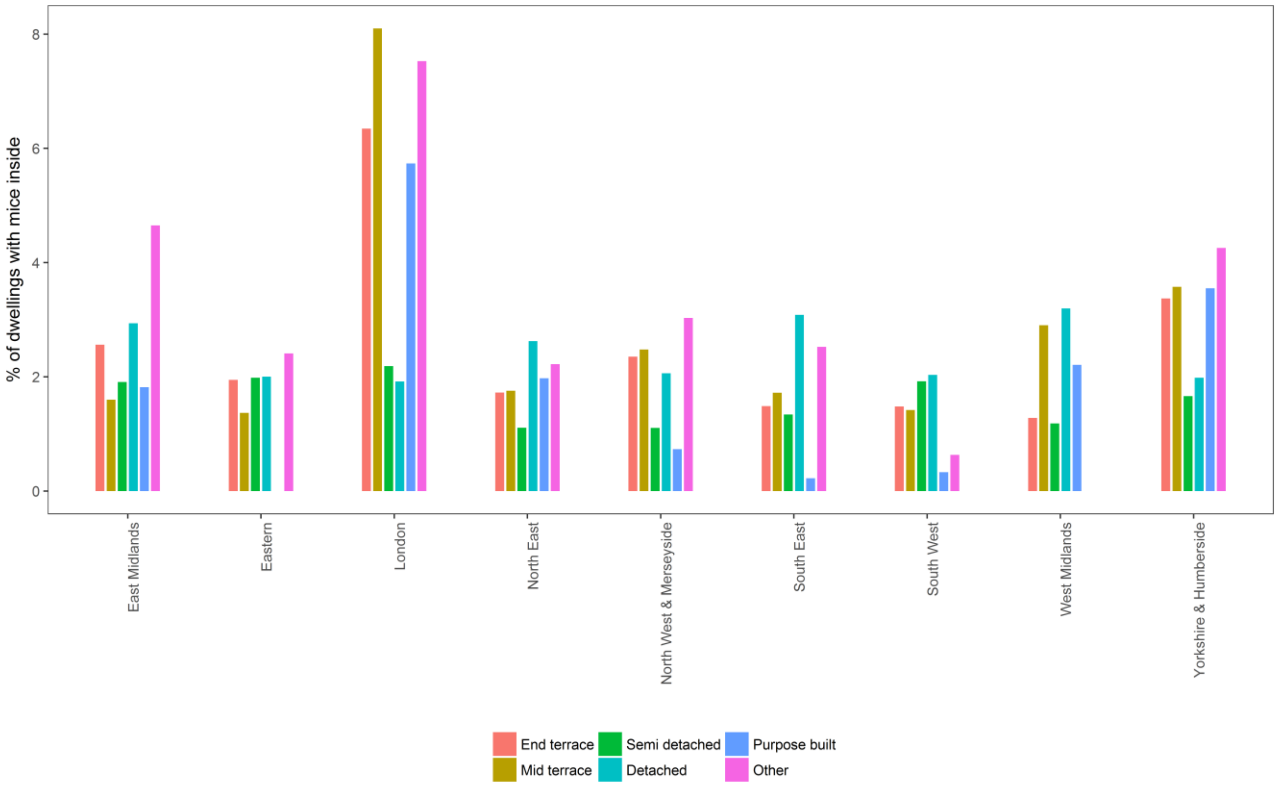

b) Tenure type

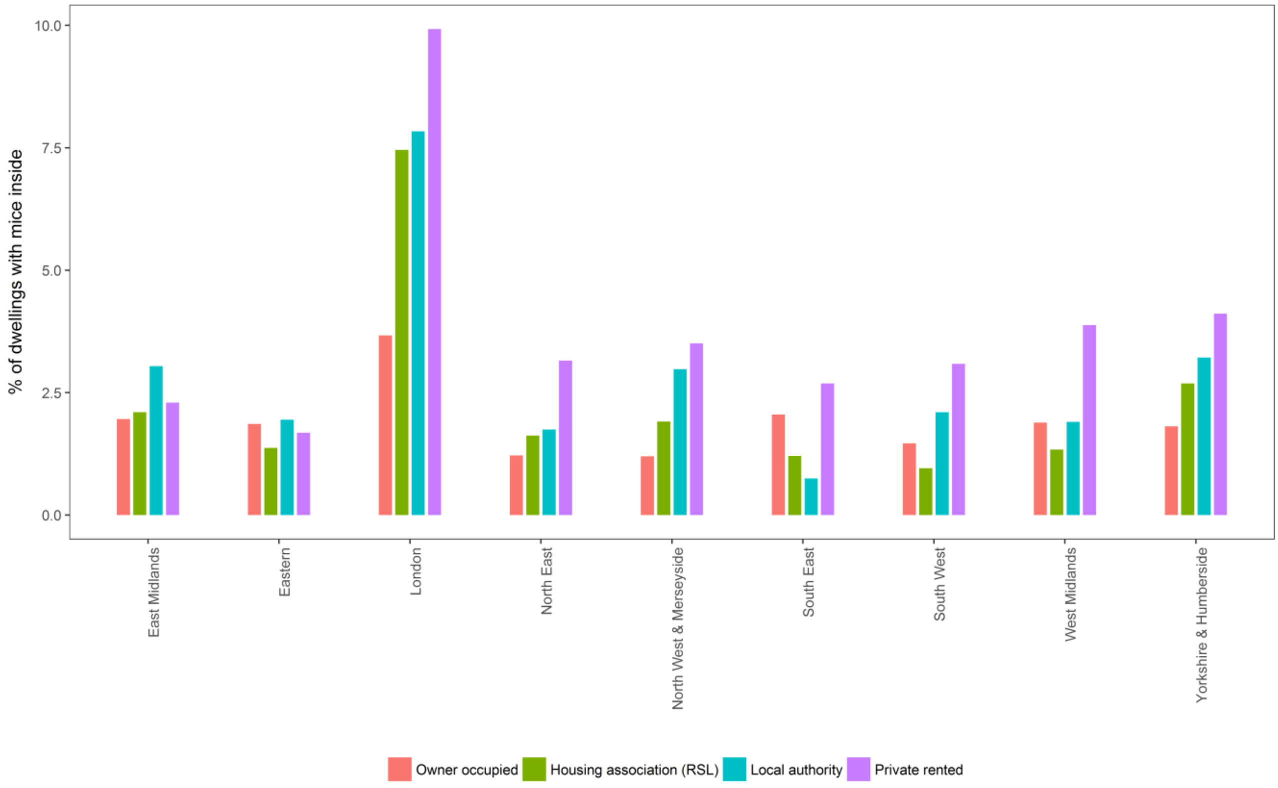

c) Problems in area

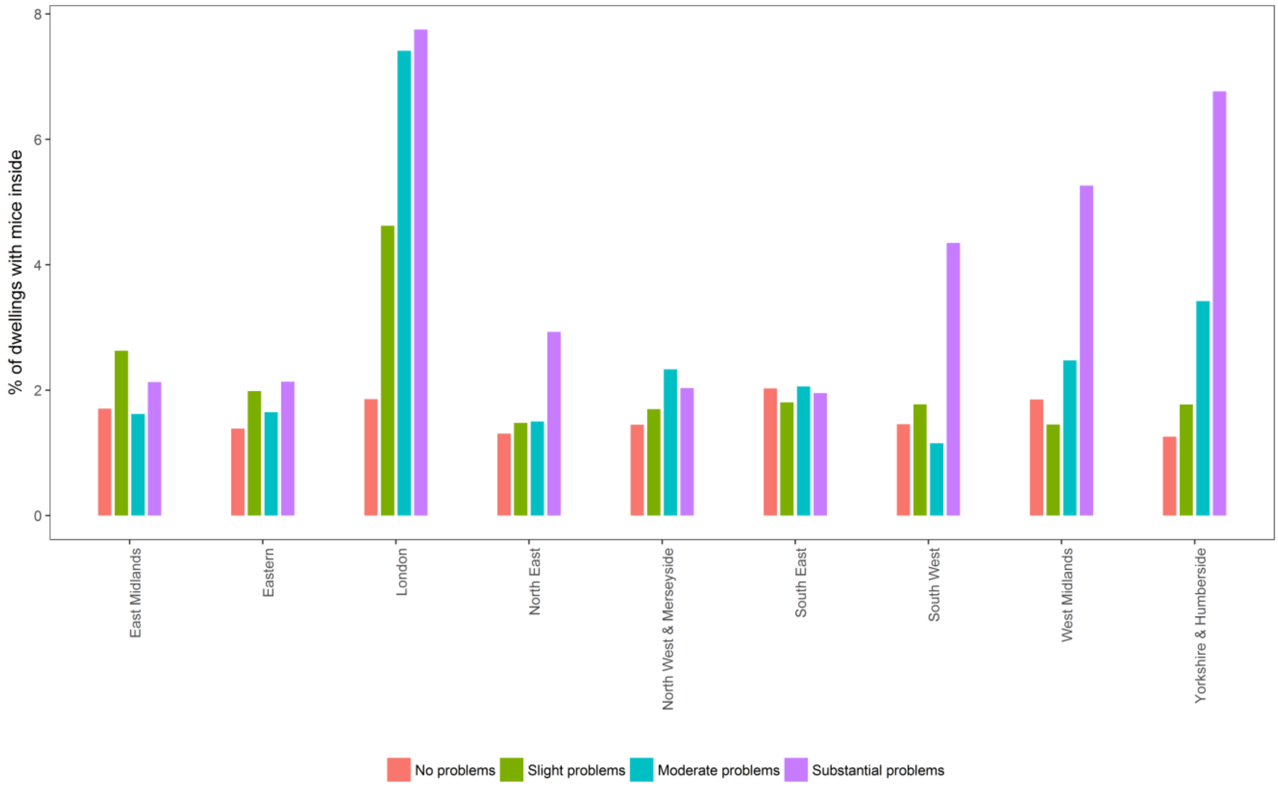

Supplementary Figure S3: Statistical significant ($p<0.05$) interactions between the variable region and other model variables on the prevalence of rats inside dwellings

a) Litter

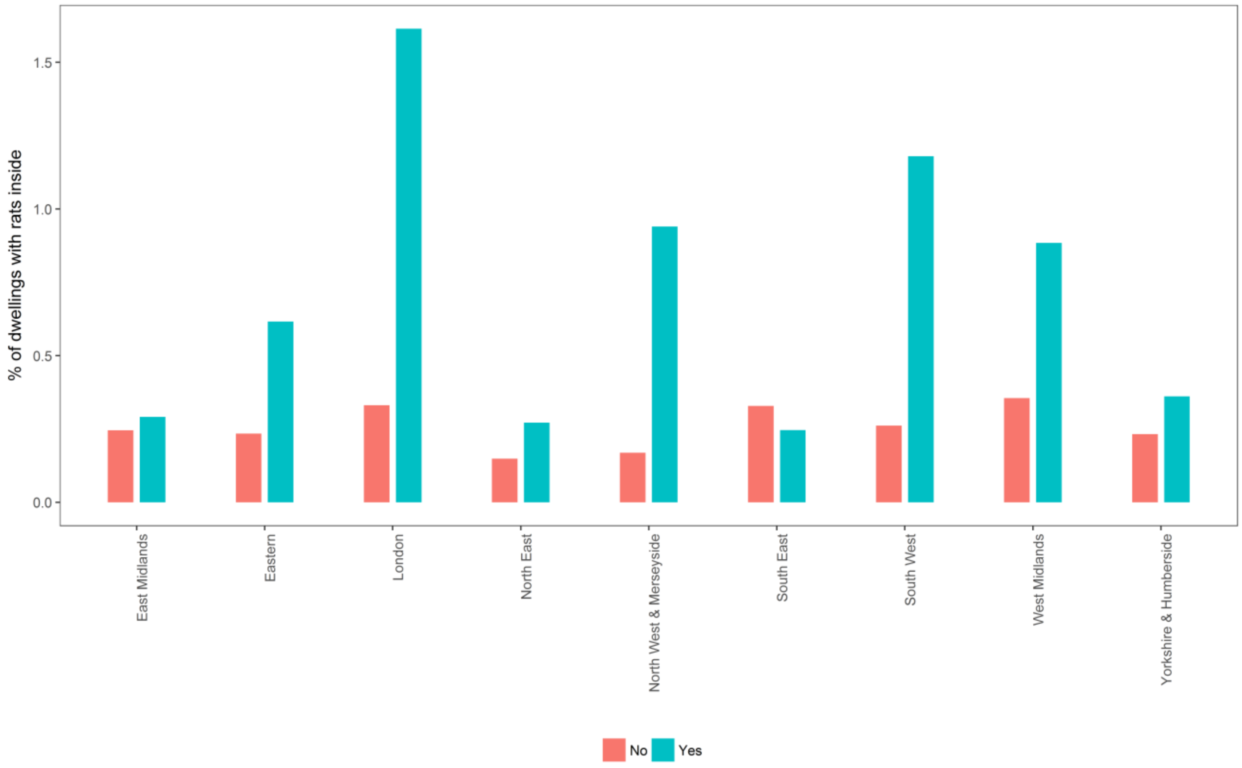

b) Plot width

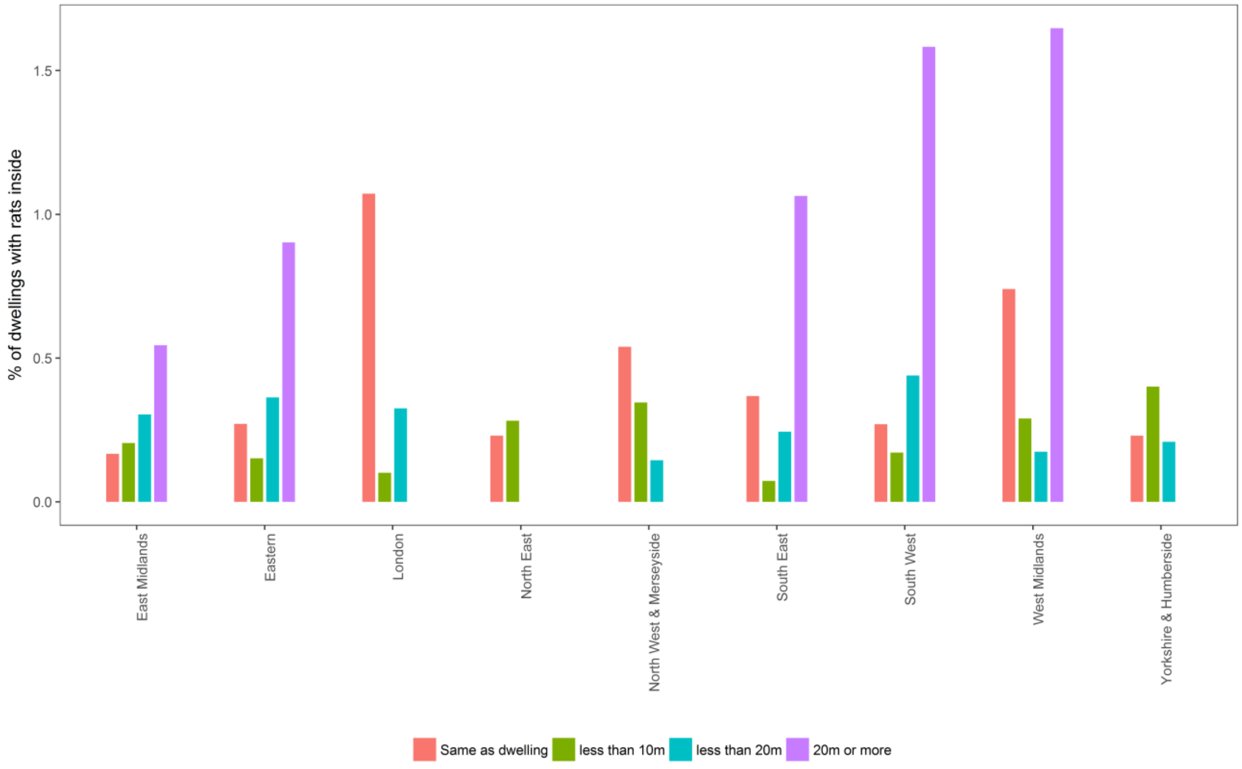

Supplementary Figure S4: Statistical significant ($p<0.05$) interactions between the variable region and other model variables on the prevalence of rats outside dwellings

a) Drainage system faults

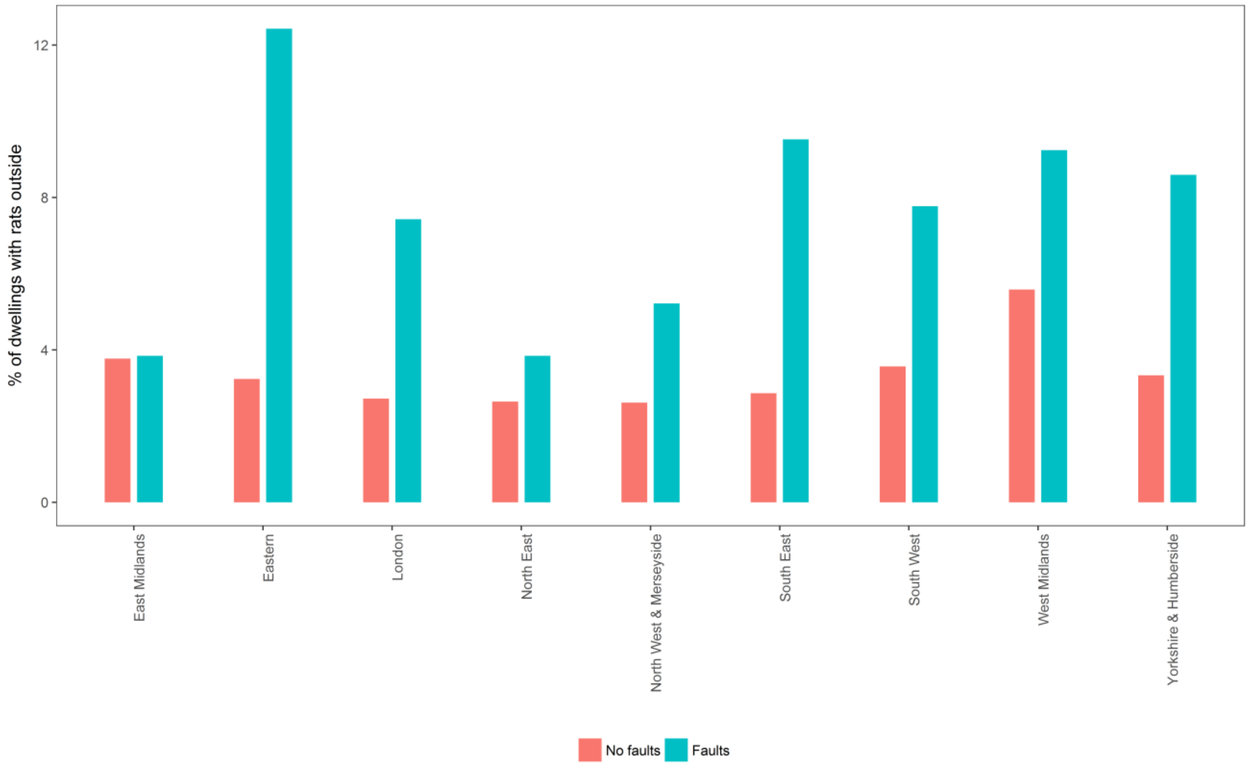

b) Number of dwellings in area

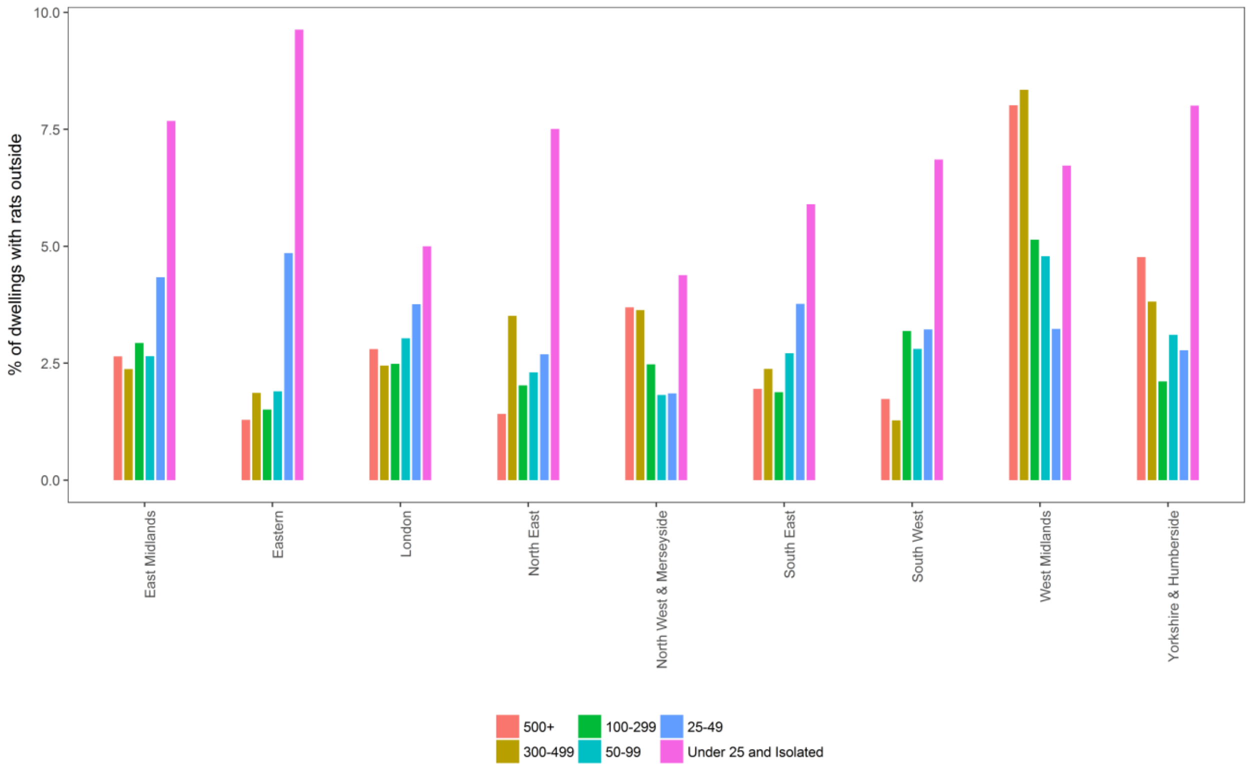

c) Nature of area

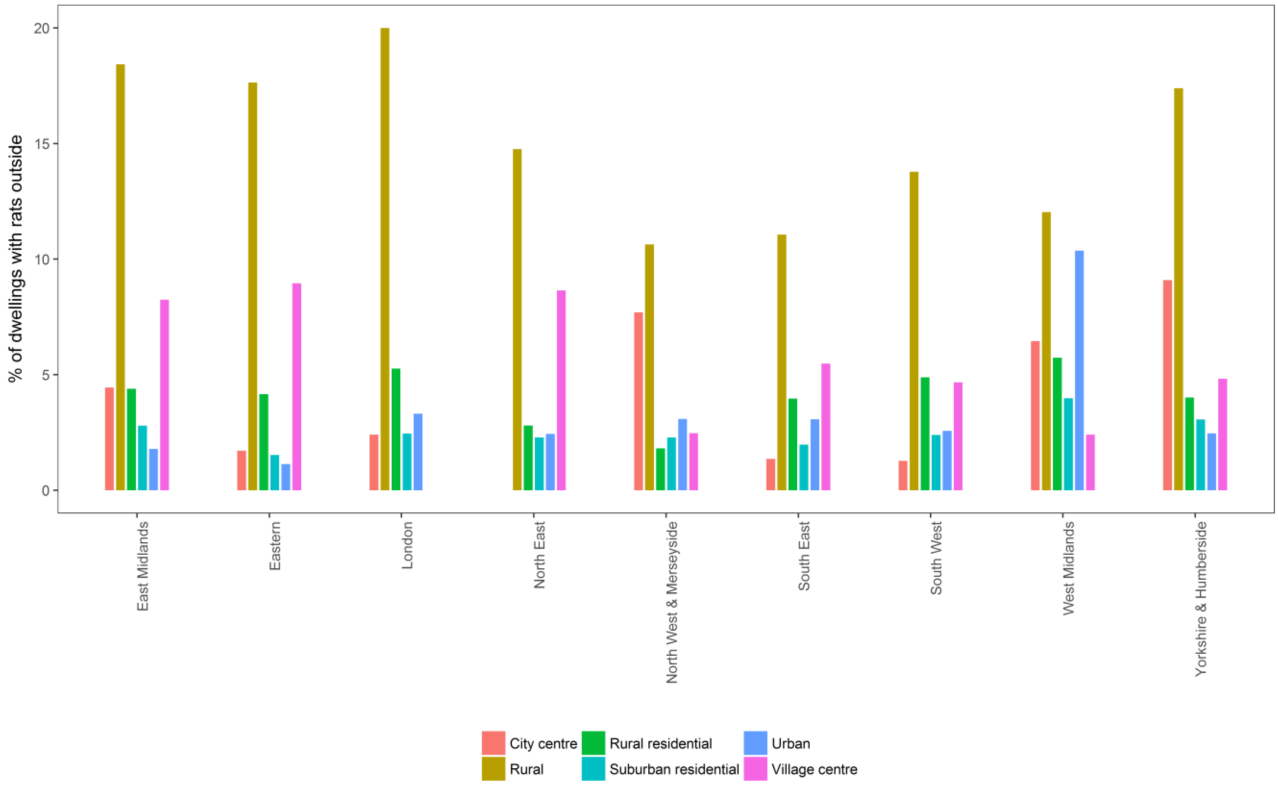

d) Problems in area

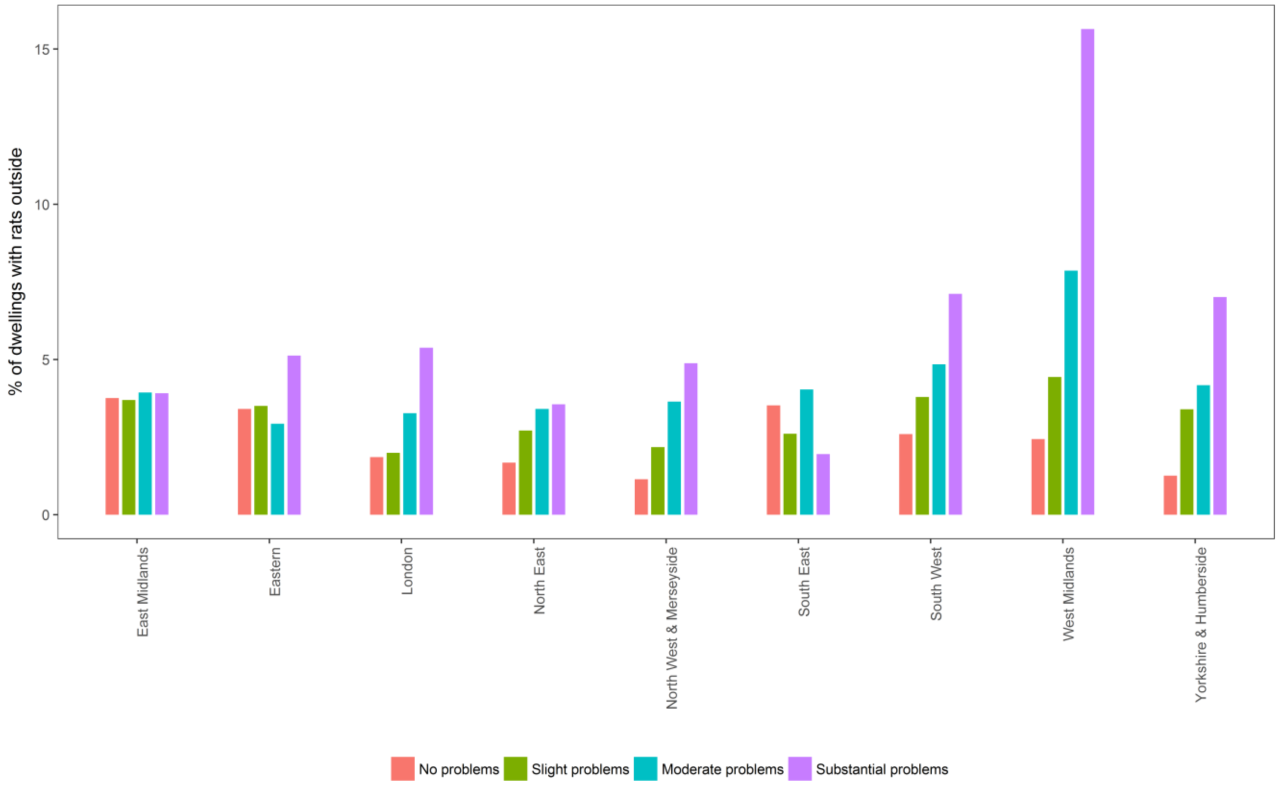

Supplementary Figure S5: Temporal variations in the percentage of dwellings surveyed tabulated across 12 variables

a) Litter

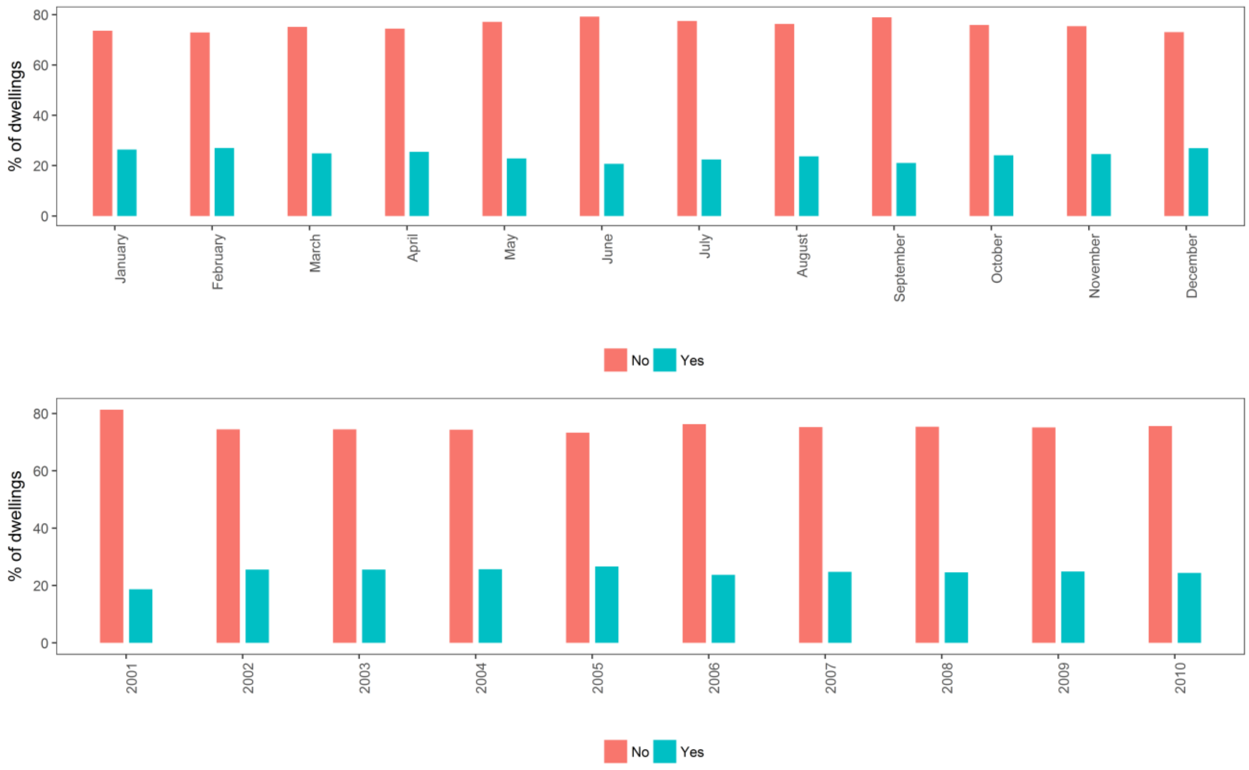

b) Number of dwellings in area

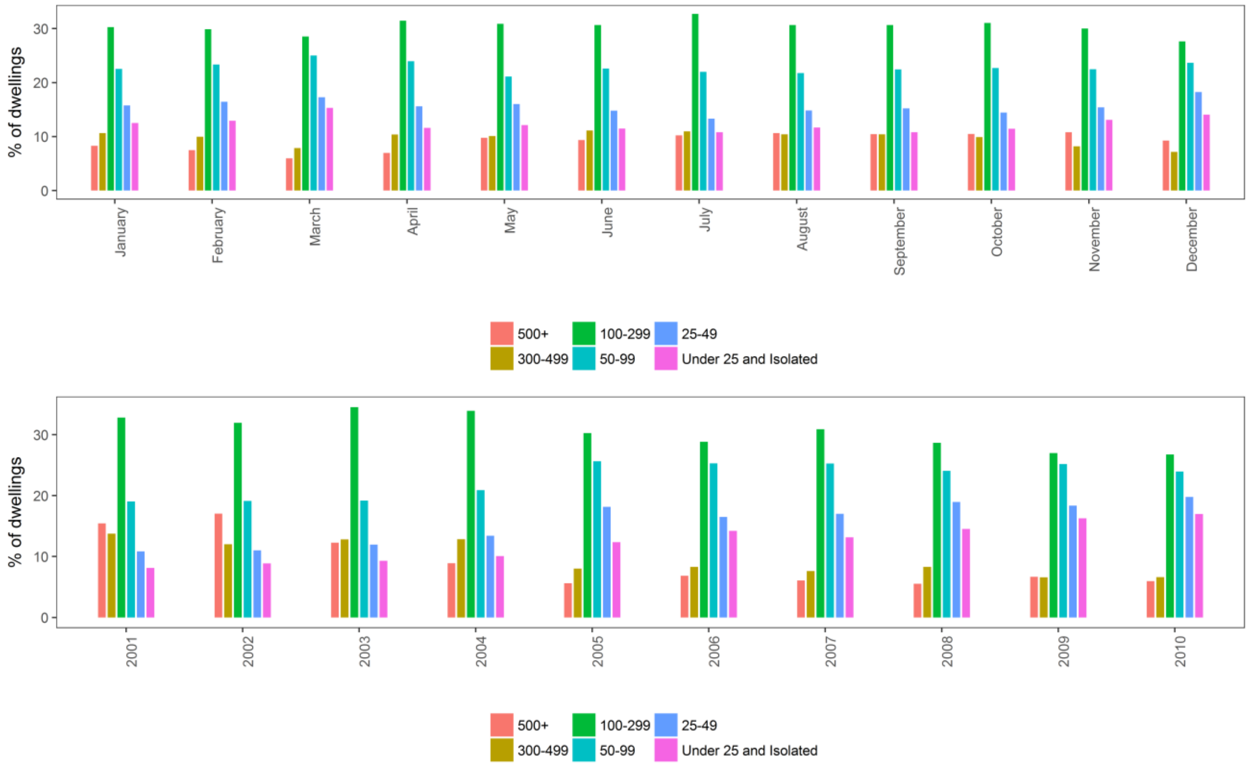

c) Pets/livestock kept outside

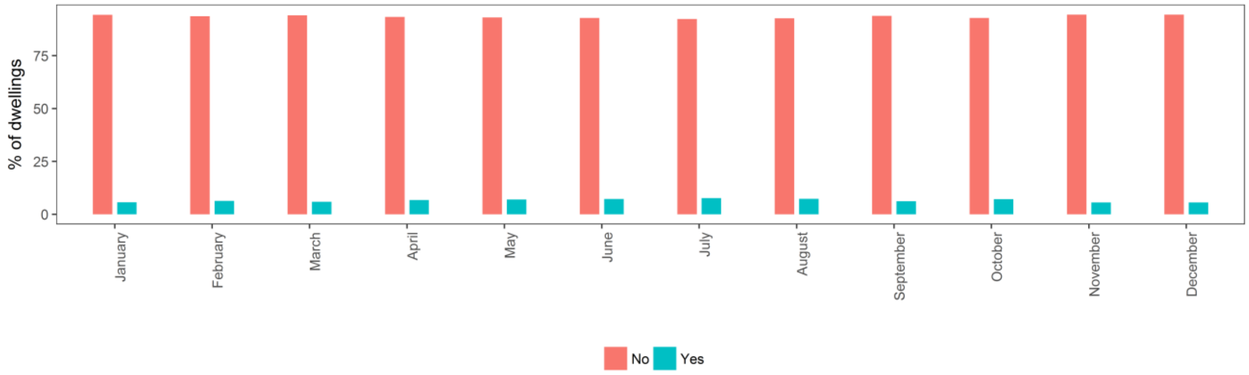

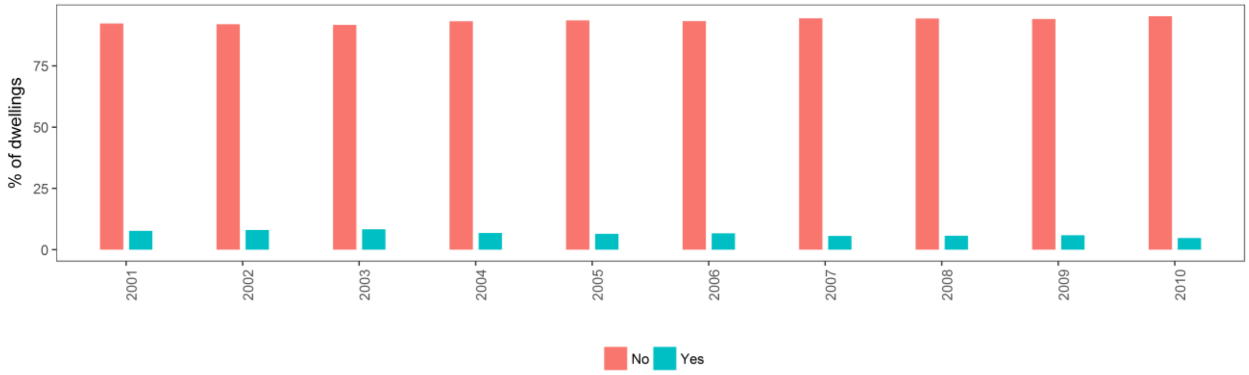

d) Plot width

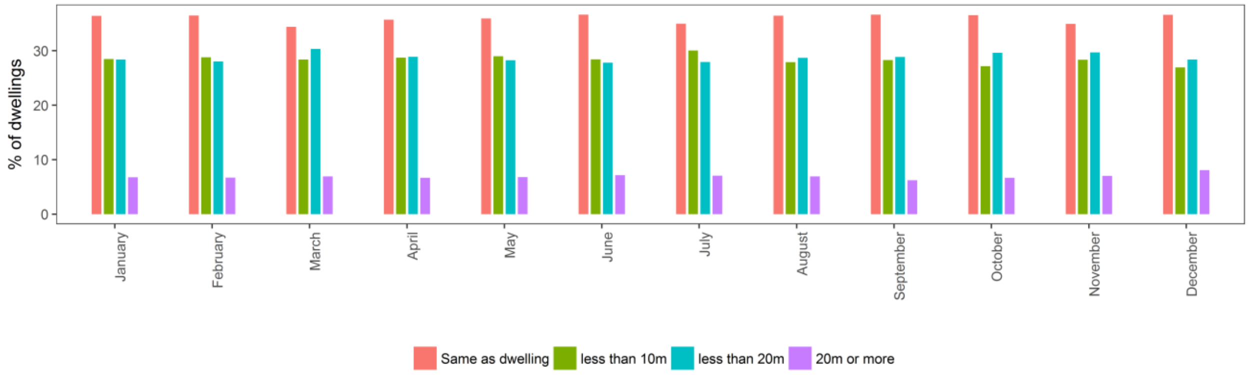

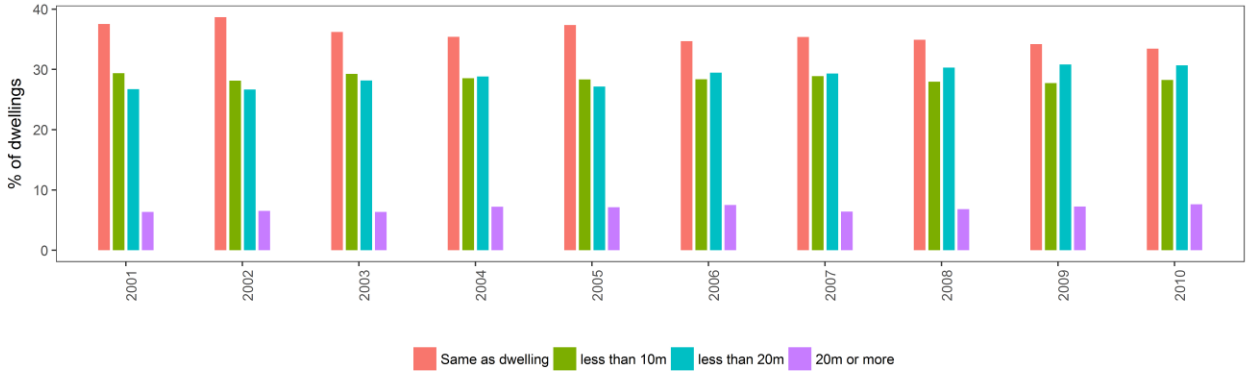

e) Road type

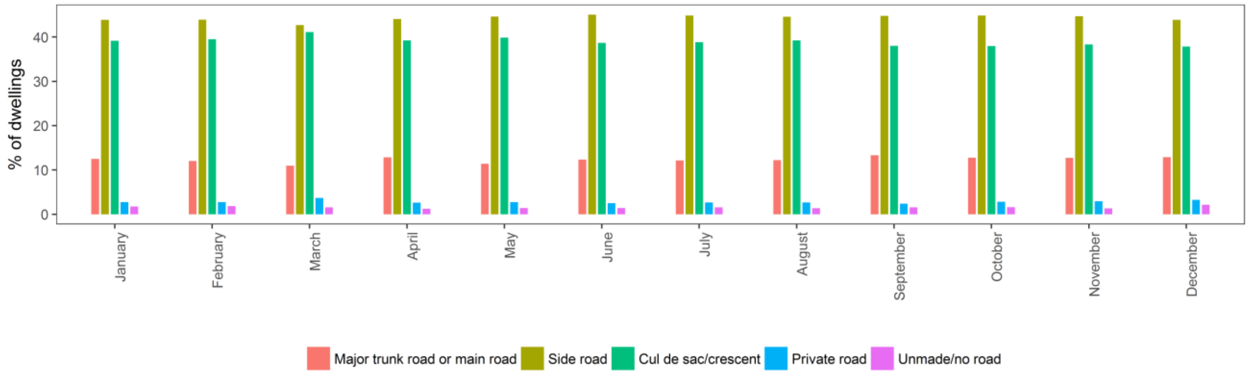

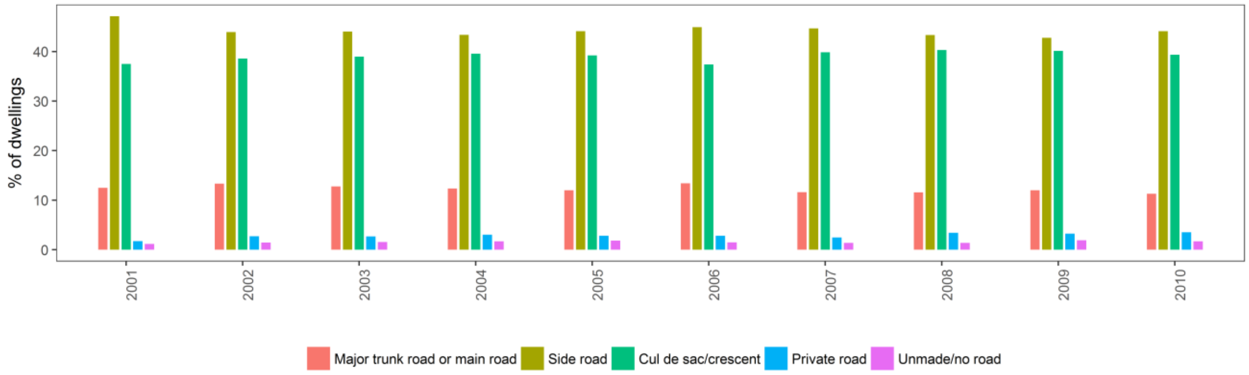

f) Nature of area

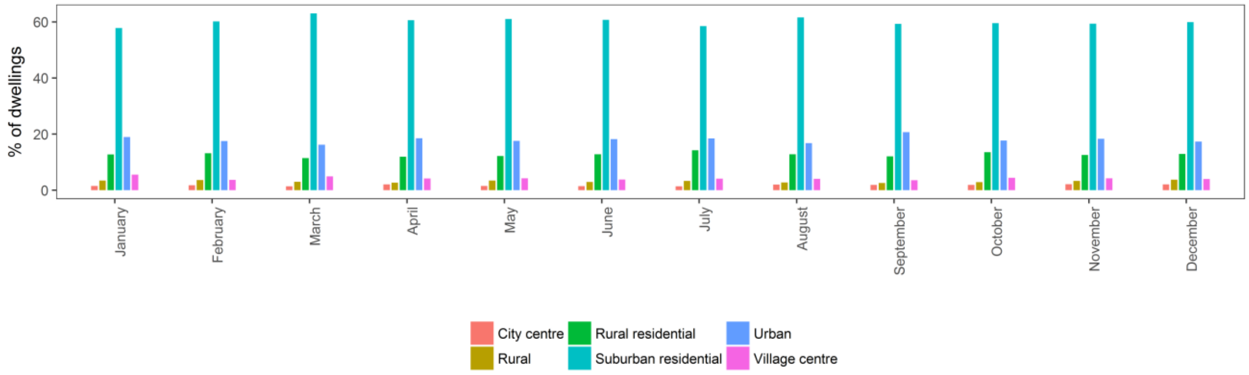

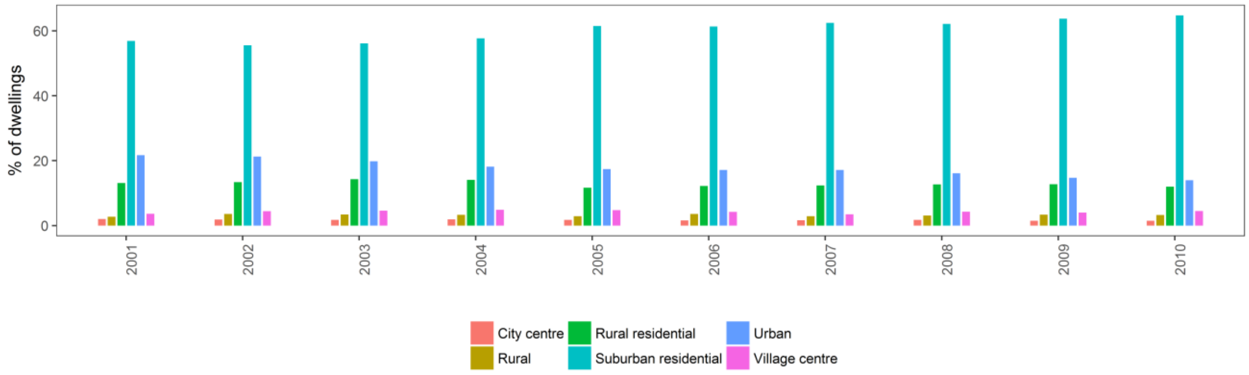

g) Drainage system faults

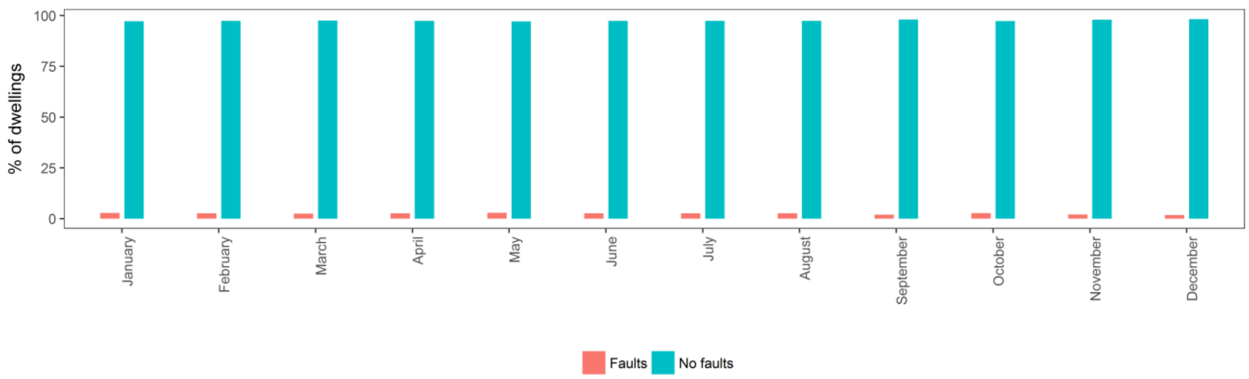

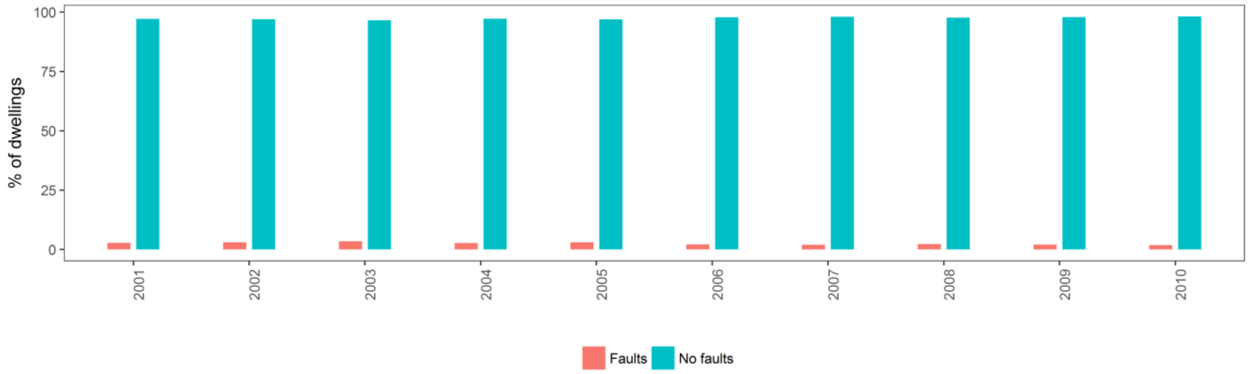

h) Problems in area

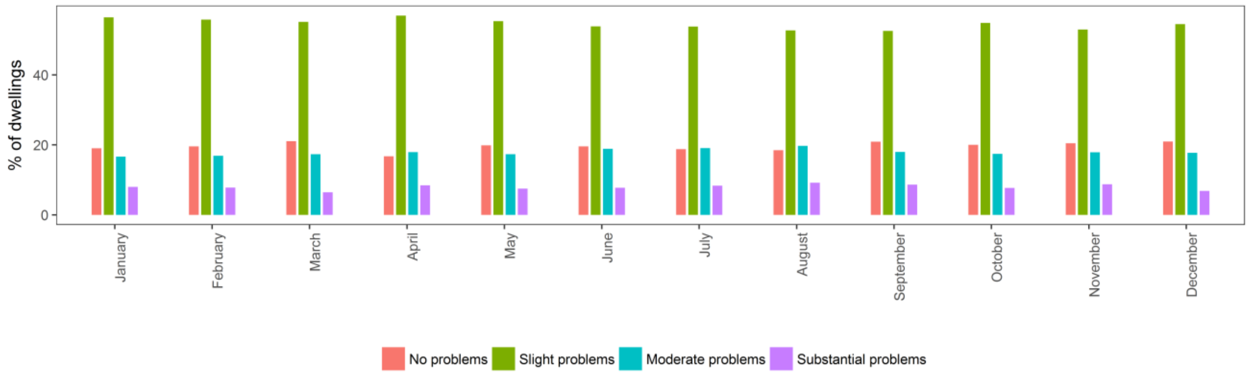

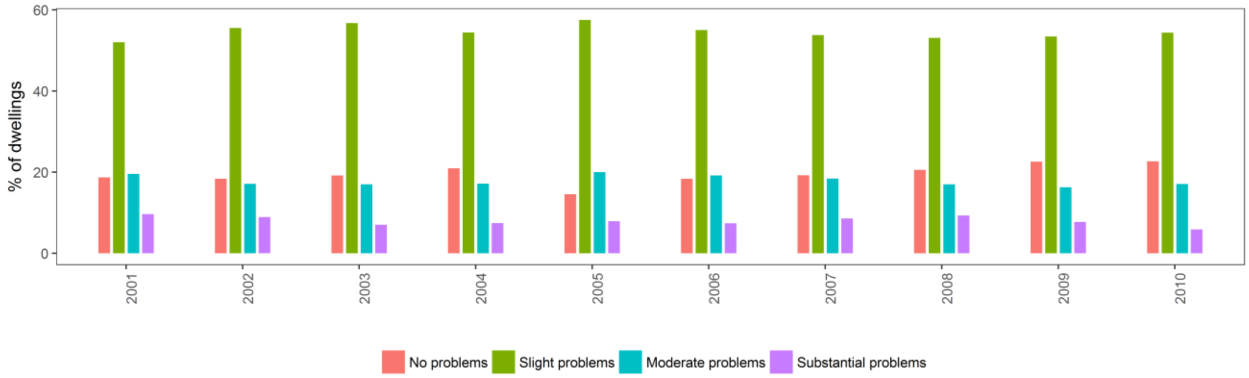

i) Tenure type

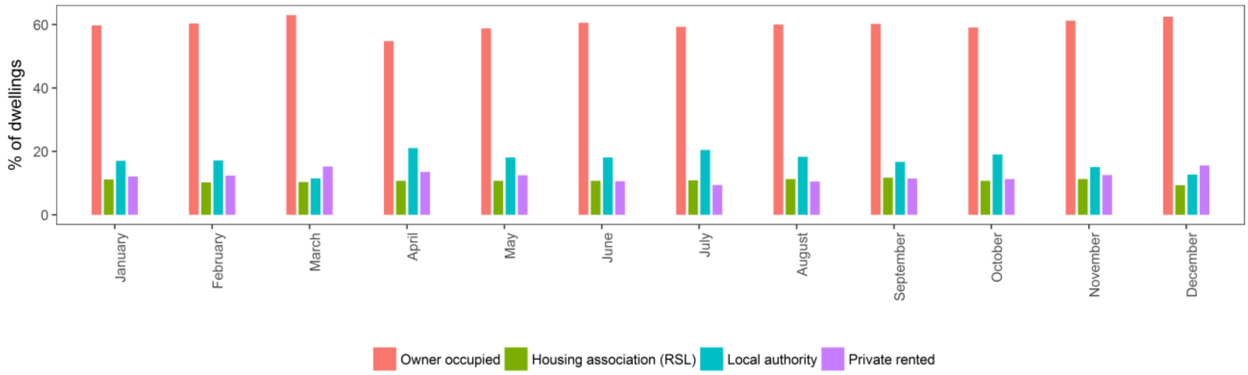

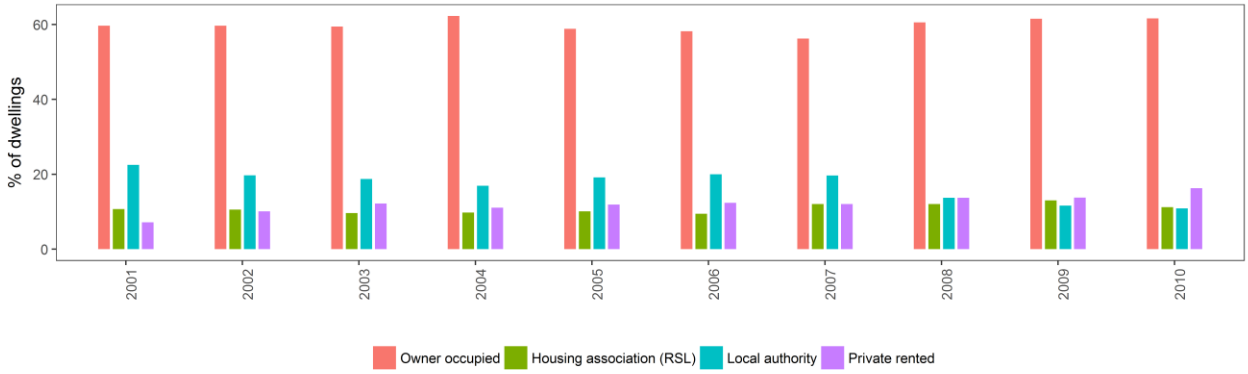

j) Dwelling type

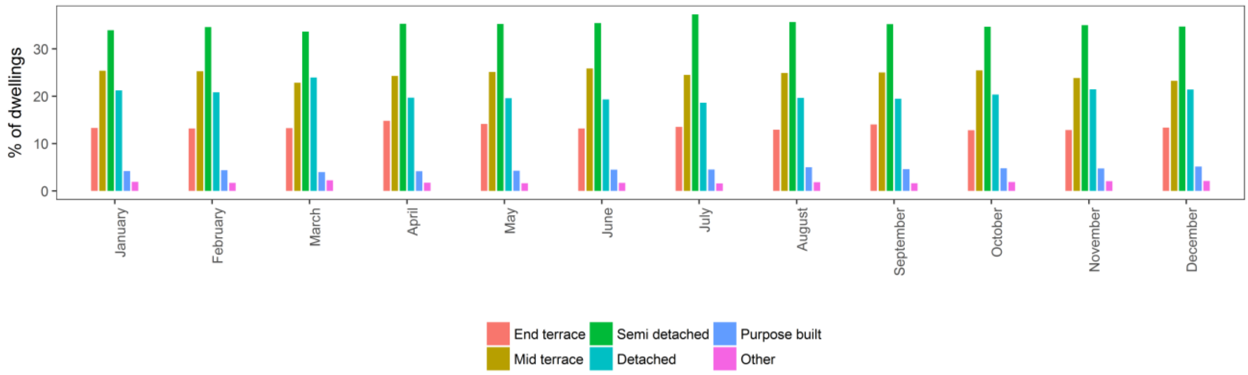

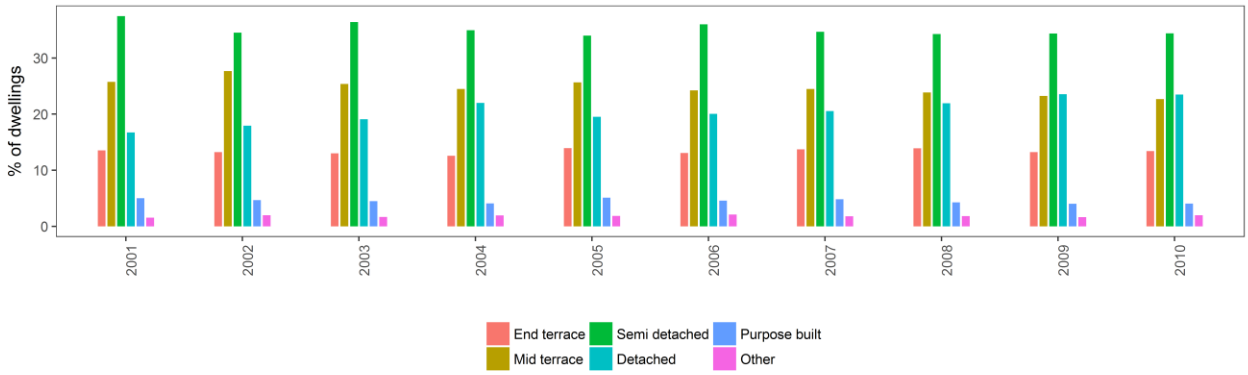

k) Date of construction

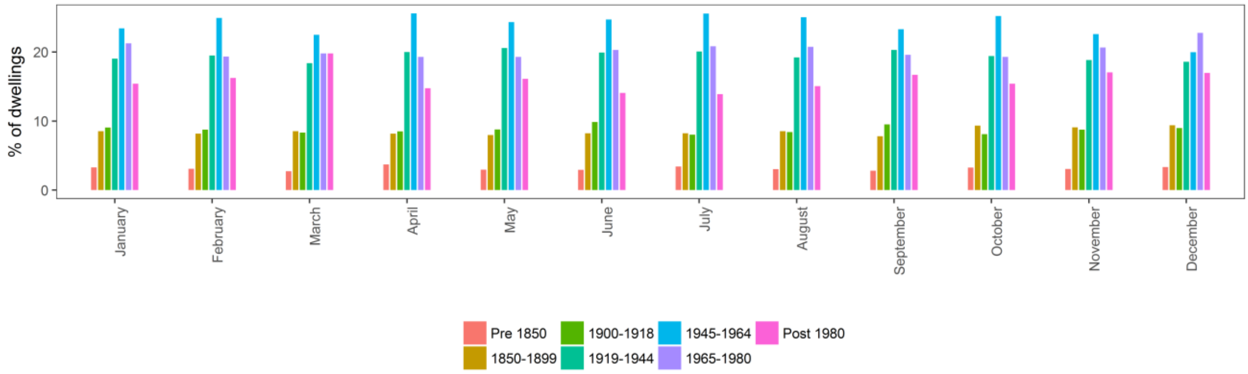

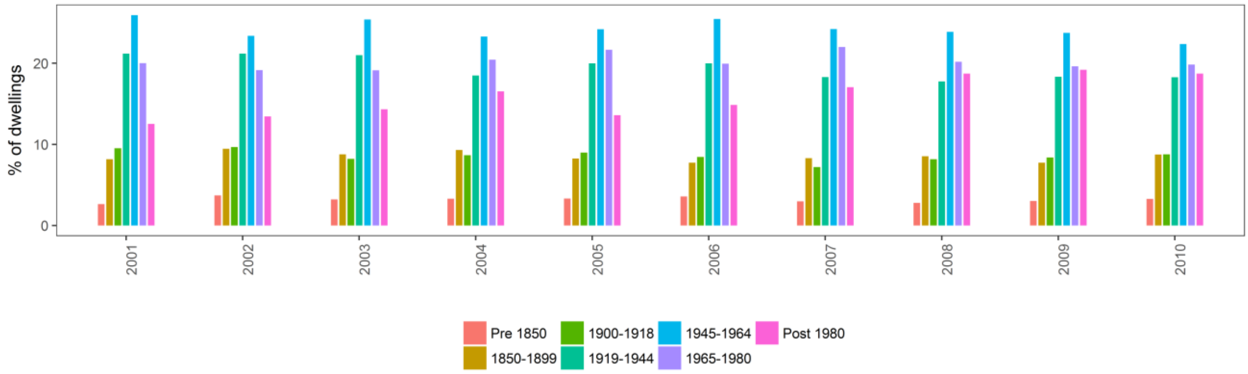

l) Region

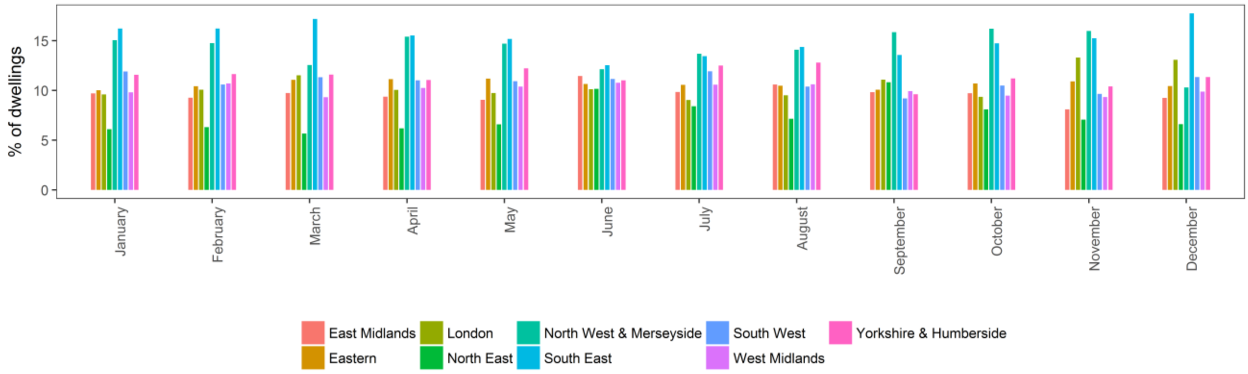

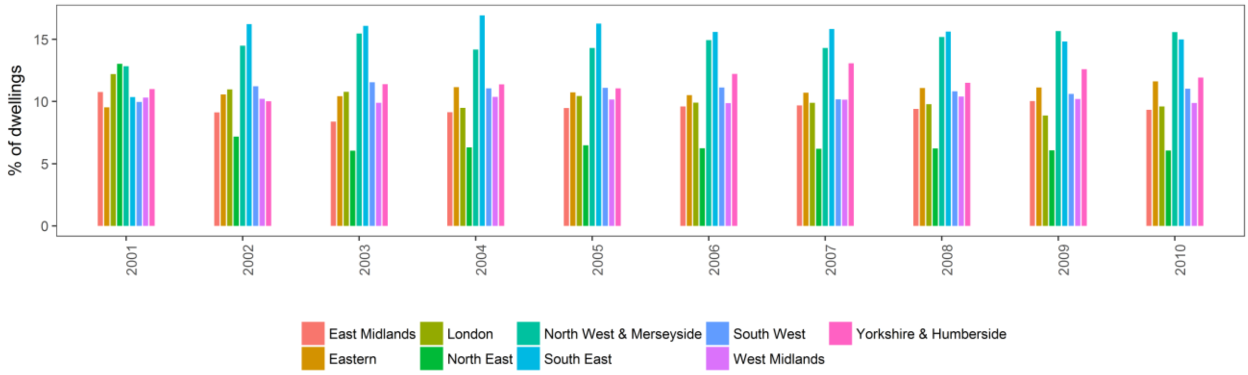

Supplementary Figure S6: Temporal variation in the percentage of dwellings with a current rodent problem (and where information on rodent control arrangements was recorded) having a) done something about the problem and b) taking action at the time of the survey according to their tenure type.

a)

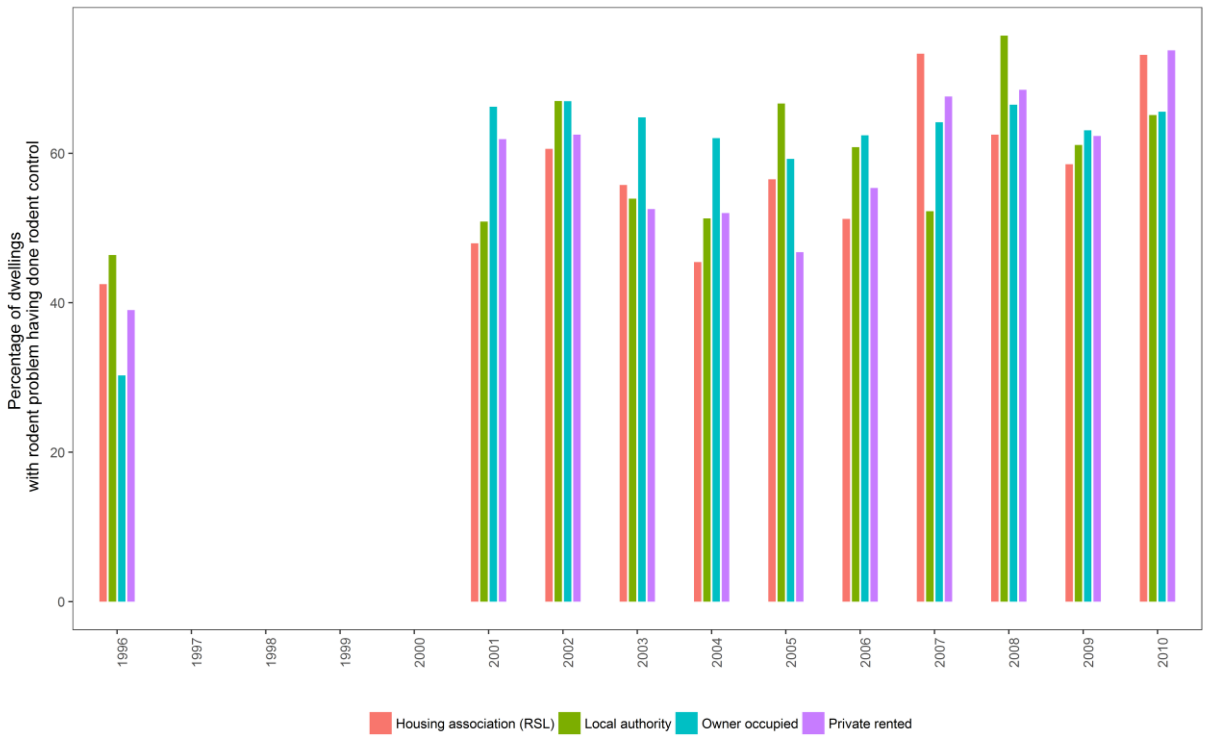

b)

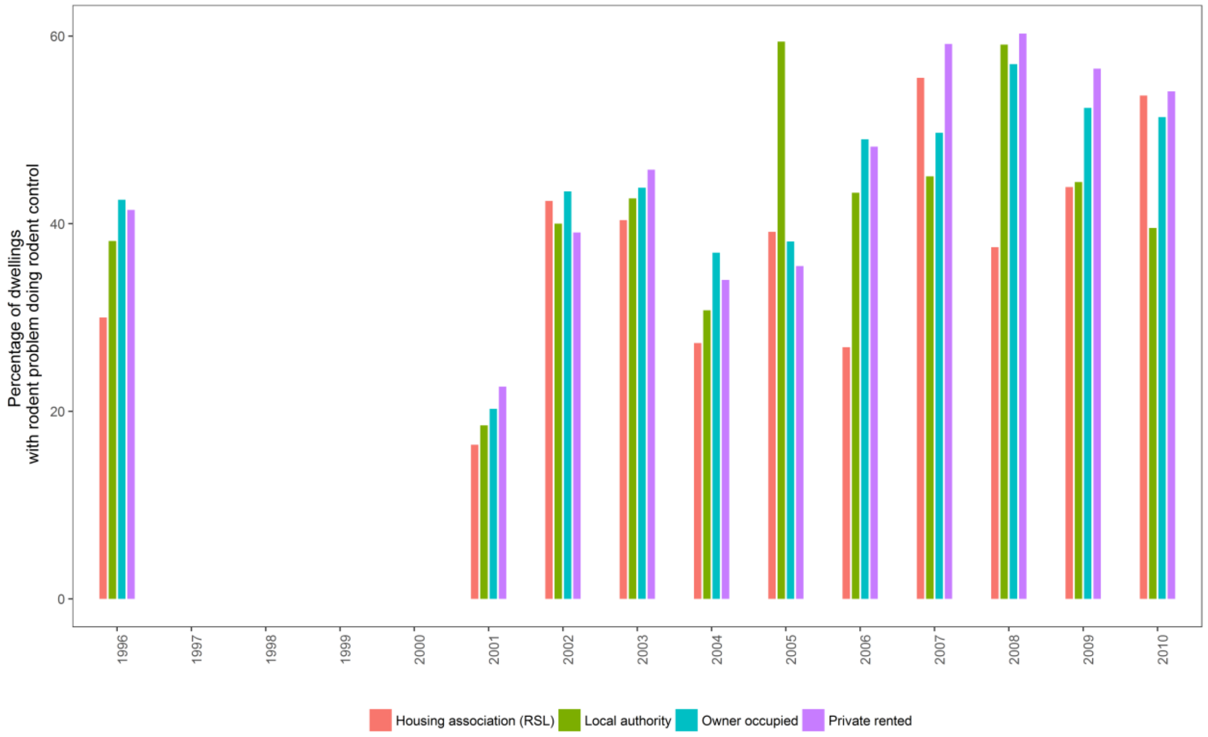

Supplementary Figure S7: Estimated smoothed function, using thin plate regression splines, for year of survey on the percentage of dwellings with a current rodent problem (and where information on rodent control arrangements was recorded) having a) done something about the problem and b) taking action at the time of the survey.The solid line is the smoother and the dotted lines are the 95% confidence bands. The y-axis represents the value taken by the centered smoother. It is the contribution (at a value of the covariate) made to the fitted value for that smooth function.

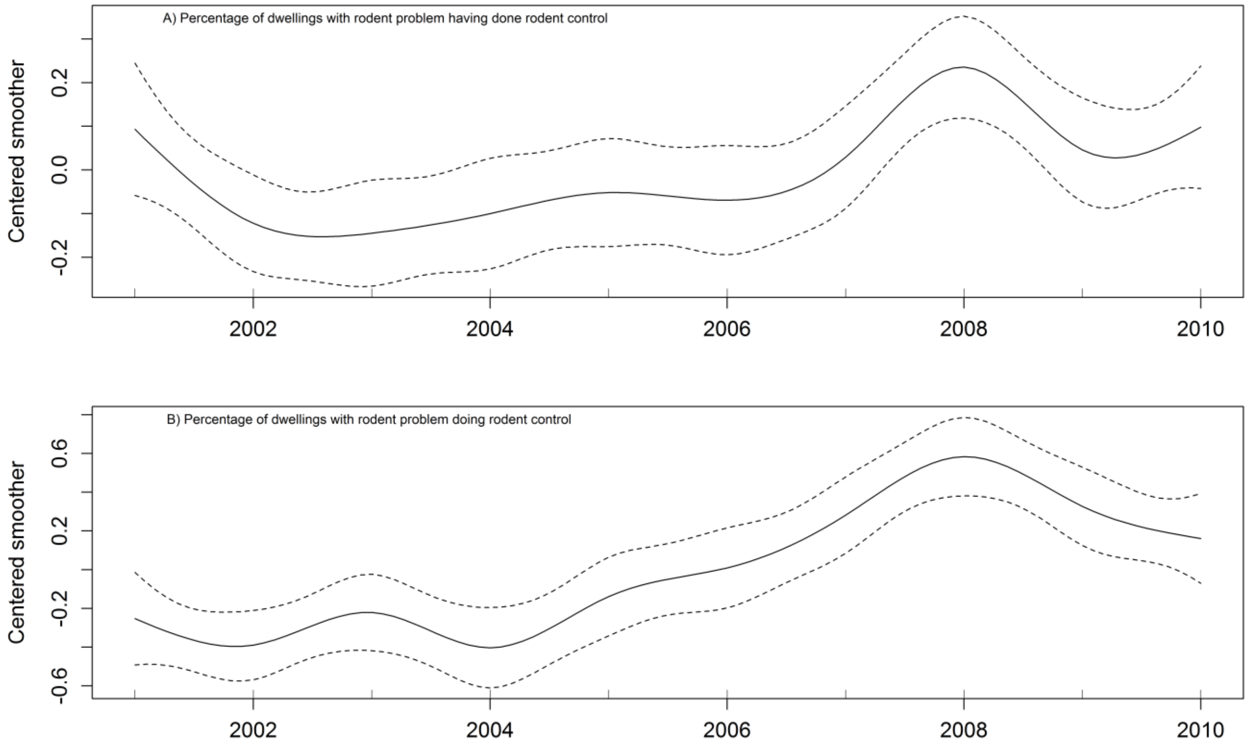

The model selection process suggested the use of generalized additive models (GAM) fitted with binomial errors and logit link function to model the effects of year of survey as a smoothed function on the percentage of dwellings with a current rodent problem (and where information on rodent control arrangements was recorded) having a) done something about the problem (df = 8.34, deviance, 30.58, p<0.001) and b) taking action at the time of the survey (df = 8.49, deviance, 79.42, p<0.001), with the estimated smoothers showing pronounced non-linear effects.

References

1. Burnham, K.P. & Anderson, D.R. *Model selection and multimodel inference: a practical information-theoretic approach*, (Springer-Verlag New York, 2002).

2. Zuur, A., Ieno, E., Walker, N., Saveliev, A. & Smith, G.C. *Mixed Effects Models and Extensions in Ecology with R*, (Springer-Verlag New York, 2009).
